# Cycloaddition and C–S Bond Cleavage Processes in Reactions of Heterometallic Phosphinidene-Bridged MoRe and MoMn Complexes with Alkynes and Phenyl Isothiocyanate

**DOI:** 10.1021/acs.organomet.3c00242

**Published:** 2023-07-07

**Authors:** M. Angeles Alvarez, M. Esther García, Daniel García-Vivó, Miguel A. Ruiz, Patricia Vega

**Affiliations:** Departamento de Química Orgánica e Inorgánica/IUQOEM, Universidad de Oviedo, E-33071 Oviedo, Spain

## Abstract

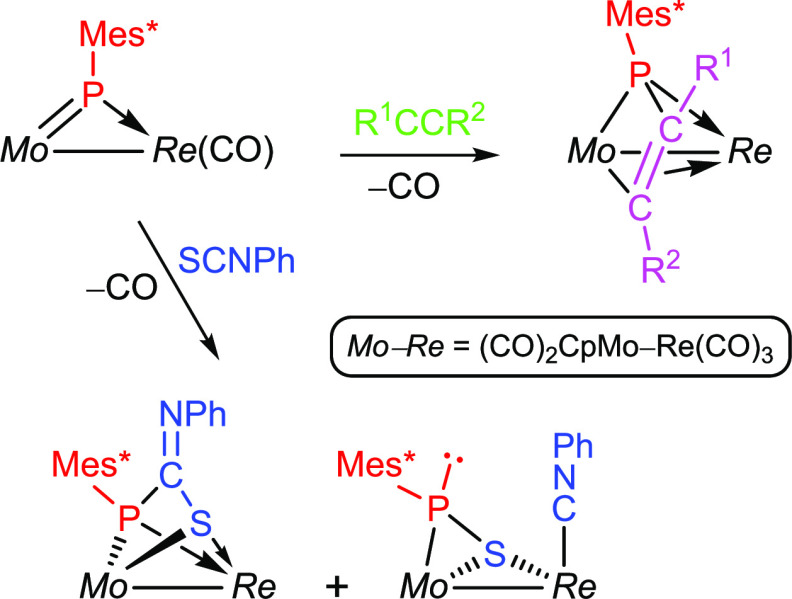

Reactions of [MoReCp(μ-PMes*)(CO)_6_] with internal alkynes RC≡CR yielded the phosphapropenylidene-bridged complexes [MoReCp(μ-κ^2^_P,C_:η^3^-PMes*CRCR)(CO)_5_] (Mes* = 2,4,6-C_6_H_2_^*t*^Bu_3_; R = CO_2_Me, Ph). Terminal alkynes HC≡CR^1^ gave mixtures of isomers [MoReCp(μ-κ^2^_P,C_:η^3^-PMes*CHCR^1^)(CO)_5_] and [MoReCp(μ-κ^2^_P,C_:η^3^-PMes*CR^1^CH)(CO)_5_], with the first isomer being major (R^1^ = CO_2_Me) or unique (R^1^ = ^*t*^Bu), indicating the relevance of steric repulsions during the [2 + 2] cycloaddition step between Mo=P and C≡C bonds in these reactions. Similar reactions were observed for [MoMnCp(μ-PMes*)(CO)_6_]. Addition of ligands to these complexes promoted rearrangement of the phosphapropenylidene ligand into the allyl-like μ-η^3^:κ^1^_C_ mode, as shown by the reaction of [MoReCp(μ-κ^2^_P,C_:η^3^-PMes*CHC(CO_2_Me)}(CO)_5_] with CN(*p*-C_6_H_4_OMe) to give [MoReCp{μ-η^3^:κ^1^_C_-PMes*CHC(CO_2_Me)}(CO)_5_{CN(*p*-CH_4_OMe)}_2_]. The MoRe phosphinidene complex reacted with S=C=NPh to give as major products the phosphametallacyclic complex [MoReCp{μ-κ^2^_P,S_:κ^2^_P,S_-PMes*C(NPh)S}(CO)_5_] and its thiophosphinidene-bridged isomer [MoReCp(μ-η^2^:κ^1^_S_-SPMes*)(CO)_5_(CNPh)]. The first product follows from a [2 + 2] cycloaddition between Mo=P and C=S bonds, with specific formation of P—C bonds, whereas the second one would arise from the alternative cycloaddition involving the formation of P—S bonds, more favored on steric grounds. The prevalence of the μ-η^2^:κ^1^_S_ coordination mode of the SPMes* ligand over the μ-η^2^:κ^1^_p_ mode was investigated theoretically to conclude that steric congestion favors the first mode, while the kinetic barrier for interconversion between isomers is low in any case.

## Introduction

**Chart 1:**
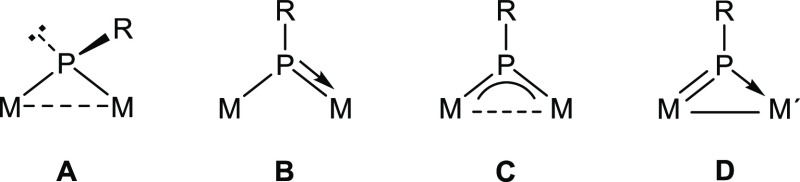
Coordination Modes of PR Ligands at Binuclear Complexes

The chemistry of mononuclear transition-metal complexes displaying phosphinidene ligands (PR) is a mature research field that has enabled the building up of a great variety of organophosphorus molecules, as a result of the high reactivity of the corresponding M–P multiple bonds toward a great diversity of small organic molecules.^1,2^ Binuclear phosphinidene-bridged complexes, on the other side, have also been found to be highly reactive toward this sort of molecules, with a chemical behavior strongly dependent on the specific coordination mode of the PR ligand (**A** to **C** in Chart 1), which enables them to render a great variety of complexes bearing bridging organophosphorus ligands in novel or rare coordination modes.^3^ This previous research, however, has been generally developed on *homometallic* complexes, and our knowledge of the chemical behavior of phosphinidene-bridged *heterometallic* complexes is very scarce. Yet, studies on other types of heterometallic complexes have revealed that the presence of distinct metal atoms, each with different electron densities and coordination spheres, produces cooperative and synergic effects that lead to singular and often increased reactivities.^4^ We thus started a study aimed at synthesizing new heterometallic PR-bridged complexes and at exploring their chemical behavior.^5−7^ An unexpected feature of several of these new complexes was the presence of a metal-phosphorus π-bonding interaction mainly located at one of the M-P junctions, in spite of identical electron count (15) of the metal fragments involved, to configure a new coordination mode of the bridging phosphinidene ligand (**D** in Chart 1). Preliminary studies on one of these species, the MoRe complex [MoReCp(μ-PMes*)(CO)_6_] (**1a**), (Mes* = 2,4,6-C_6_H_2_^*t*^Bu_3_), disclosed a particular tendency of this molecule to undergo cycloaddition processes at its Mo—P double bond when reacting with unsaturated organic molecules such as the terminal alkyne HC≡CCO_2_Me, or the isocyanide C≡N(*p*-C_6_H_4_OMe), to render novel or unusual organophosphorus ligands in new coordination modes.^6^ This was later corroborated by our full studies on the reactivity of **1a** and its MoMn analogue [MoMnCp(μ-PMes*)(CO)_6_] (**1b**) toward diazoalkanes and organic azides.^8^ In this paper, we complete our studies on the cycloaddition reactions of these heterometallic complexes by exploring in more detail the reactions of **1a,b** with different terminal and internal alkynes, as well as their reactions with phenyl isothiocyanate, an heterocumulene that might interact with these complexes through either S—C or C—N double bonds.

**Scheme 1:**
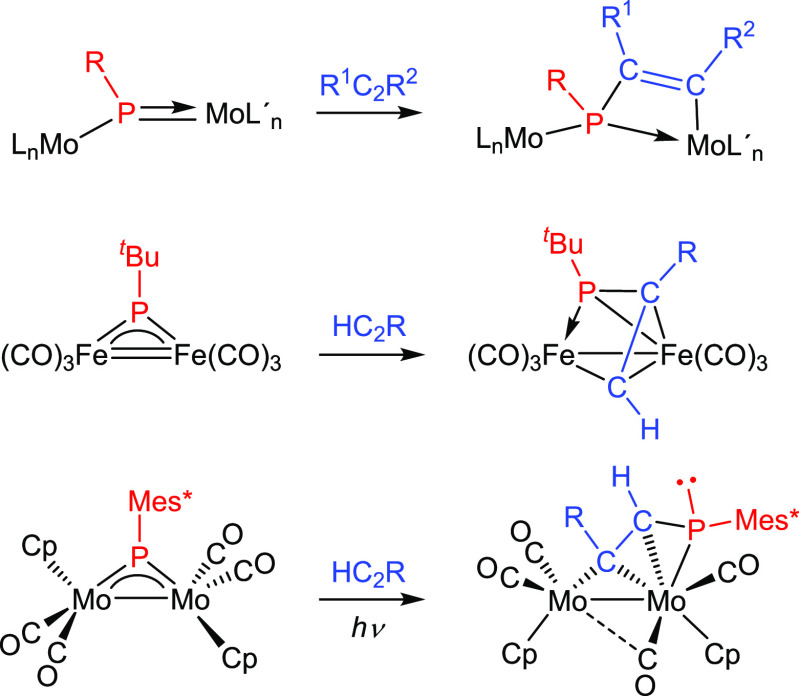
Reactions of Trigonal PR Complexes with Alkynes

The reactivity of homometallic PR-bridged complexes toward alkynes has been recently reviewed by us.^3,9^ Of particular interest to the present work are reactions of trigonal PR complexes of types **B** and **C** (Scheme 1). These reactions often result in [2 + 2] cycloaddition of the alkyne to the multiple M—P bond of the metal substrate, as observed for asymmetric Mo_2_ complexes of type **B**.^10^ This in turn may be followed by coordination of the C–C double bond of the resulting phosphametallacycle to the second metal center, as observed for [Fe_2_(μ-P^*t*^Bu)(CO)_6_], a transient and unsaturated complex of type **C**.^11^ Yet, another possible output of these reactions is full insertion of the alkyne into one of the M–P bonds, as observed in the photochemical reactions of the type **C** complex [Mo_2_Cp_2_(μ-PMes*)(CO)_4_].^9^ Most of these reactions are regioselective when using terminal alkynes, with the terminal carbon being usually the most favored site for P–C coupling (not in the mentioned Fe_2_ complex). In contrast to these relatively simple processes, the reactions of the cyclopentadienylphosphinidene complex [W_2_(μ-PC_5_Me_5_)(CO)_10_] with alkynes were extremely complex, due to the involvement of the C_5_Me_5_ group in all reactions (migration to the metal, coupling to alkynes, etc.), as shown by detailed studies by Scheer and co-workers.^12^ On the other hand, complexes of type **A**, bearing pyramidal PR ligands, may behave in even more complex ways,^3^ and they will not be summarized here. As we will discuss below, the alkyne derivatives of compounds **1a,b** follow from cycloaddition processes analogous to those observed for trigonal PR complexes of type **B,** but easy decarbonylation of the M(CO)_4_ fragments force rearrangements of the resulting metallacycles.

**Scheme 2:**
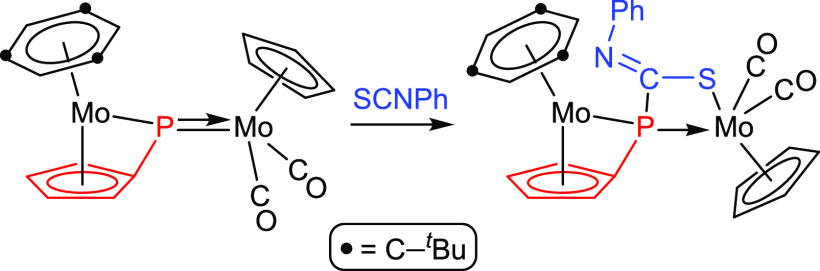
SCNPh Cycloaddition to a PR Complex of Type B

The reactions of heterocumulenes such as organic isothiocyanates (S=C=NR) with PR-bridged complexes could be in principle even more complex, since both the S–C and the N–C double bonds might be involved in the interaction with the bridging phosphinidene ligand, as noted above. However, previous knowledge of this sort of reaction is very limited, as it has been actually studied only in the case of the type **B** dimolybdenum complex [Mo_2_Cp(μ-κ^1^:κ^1^,η^5^-PC_5_H_4_)(CO)_2_(η^6^-Mes*H)] (Scheme 2). The latter complex reacts at 363 K with phenyl isothiocyanate to give a product resulting from the [2 + 2] cycloaddition between the S—C double bond of the organic reagent and the Mo–P double bond of the dimetal complex, with specific formation of a new P–C bond.^13^ We note that this is a process analogous to those observed in the reactions of phosphanyl complexes [ML(PR_2_)(CO)_2_] with different isothiocyanates (M = Mo, W; L = Cp, Cp*; R = Ph, H, ^*t*^Bu).^14^ As it will be shown below, reactions of the heterometallic complexes **1a,b** with phenyl isothiocyanate seem to involve related cycloadditions but are then followed by additional rearrangements derived from the presence of the M(CO)_4_ fragment, which again provides an additional coordination site upon decarbonylation.

## Results and Discussion

### Reactions of Compounds 1 with Alkynes

**Scheme 3:**
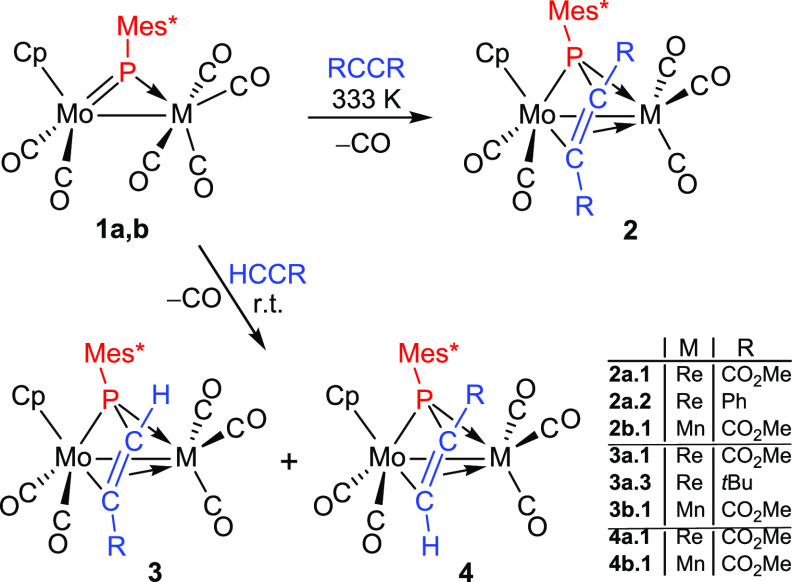
Reactions of Compounds **1** with Alkynes

**Scheme 4:**
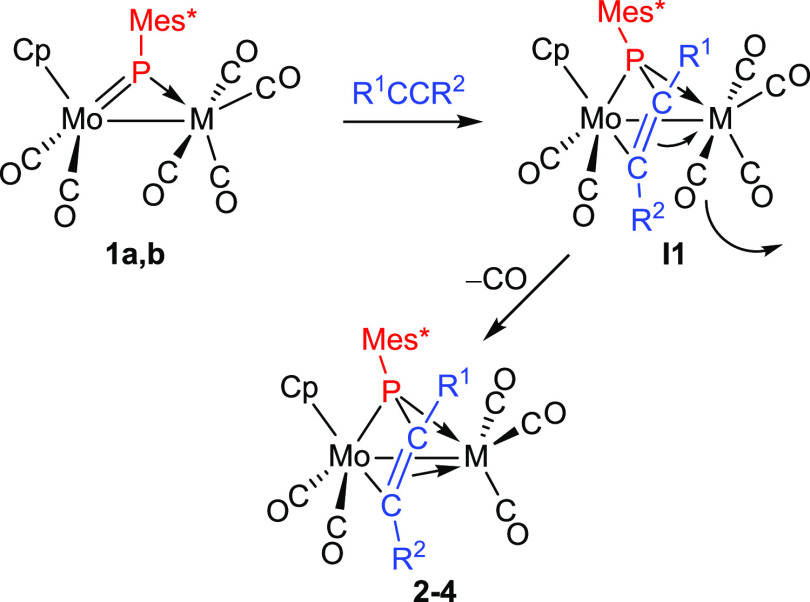
Reaction Path in the Formation of Compounds **2–4**

The rhenium compound **1a** failed to react with internal alkynes such as dimethyl acetylenedicarboxylate (DMAD) or diphenylacetylene at room temperature, but these reactions proceeded smoothly upon moderate heating (333 K) to give the phosphapropenylidene-bridged complexes [MoReCp(μ-κ^2^_P,C_:η^3^-PMes*CRCR)(CO)_5_] [R = CO_2_Me (**2a.1**), Ph (**2a.2**)], which could be isolated in ca. 70% yield as yellow solids upon chromatographic workup (Scheme 3). A similar reaction was observed between the manganese compound **1b** and DMAD, to give the analogous product [MoMnCp{μ-κ^2^_P,C_:η^3^-PMes*C(CO_2_Me)C(CO_2_Me)}(CO)_5_] (**2b.1**). The formation of all these complexes, which display a coordination of the organophosphorus ligand comparable to the one observed in the diiron complex depicted in Scheme 1, can be understood as stemming from an initial [2 + 2] cycloaddition between C≡C and Mo=P multiple bonds followed by decarbonylation of the M(CO)_4_ fragment in the resulting phosphametallacyclic intermediate **I1**; this induces the coordination of its C–C double bond to the group 7 metal atom, to yield the final product (Scheme 4). We note that the first step thus would be reminiscent of the reactions observed between alkynes and mononuclear PR complexes of nucleophilic type.^2^

In our preliminary study on the reactivity of **1a**, we found that this complex reacted with a terminal alkyne such as methyl propiolate faster than observed for the above reactions with internal alkynes, since the process was completed at room temperature in about 2 h.^6^ A similar result has been now obtained when using the manganese compound **1b**, with this reaction being completed in ca. 4 h. In both cases, a mixture of phosphapropenylidene-bridged isomers [MoMCp(μ-κ^2^_P,C_:η^3^-PMes*CHC(CO_2_Me)}(CO)_5_] [M = Re (**3a.1**), Mn (**3b.1**)] and [MoMCp(μ-κ^2^_P,C_:η^3^-PMes*C(CO_2_Me)CH}(CO)_5_] [M = Re (**4a.1**), Mn (**4b.1**)] is obtained (Scheme 3), which can be resolved through chromatographic or crystallization workup. Isomers **3** and **4** have a structure similar to compounds **2**, and they differ from each other in the identity (terminal or internal) of the carbon bound to phosphorus during the cycloaddition process. We note that in both cases the major isomer **3** obtained is the one involving P–C bond formation with the terminal carbon of the alkyne, likely more favored on steric grounds, although the relative proportions are also dependent on the metal, since the **3**/**4** ratios are ca. 5 for the rhenium complex and 2 for the manganese one (see the Experimental section). Further indication of the relevance of steric factors in the above reactions was obtained from the reaction of **1a** with *t*-butylacetylene, which proceeded slowly at room temperature to give complex [MoReCp(μ-κ^2^_P,C_:η^3^-PMes*CHC(^*t*^Bu)}(CO)_5_] (**3a.3**) as the unique product.

### Structure of the Phosphametallacyclic Complexes **2** to **4**

**Figure 1:**
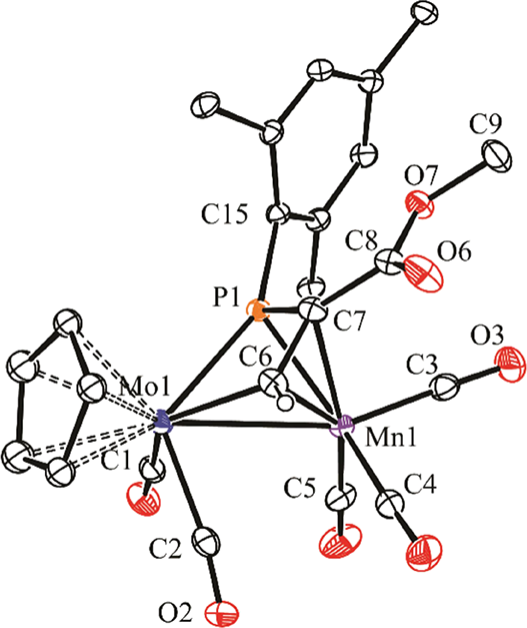
ORTEP diagram (30% probability) of compound **4b.1**, with ^*t*^Bu (except their C^1^ atoms) and most H atoms omitted for clarity.

During our preliminary exploration of the reactivity of **1a**, we determined the solid-state structure of isomer **3a.1**.^6^ We have now determined the structure of the manganese isomer **4b.1** (Figure 1 and Table 1), which expectedly displays geometrical parameters comparable to those of **3a.1**, after allowing for the differences in the covalent radii of Mo, Re, and Mn atoms.^15^ The main difference concerns the position of the CH carbon in the bridging phosphapropenylidene ligand, which is bound to phosphorus (and Re) in **3a.1**, but bridging both metal atoms in **4b.1**, in a rather symmetrical way (Mo–C6 = 2.167(3), Mn–C6 = 2.083(3) Å). The internal carbon is just coordinated to Mn, with a Mn–C separation longer than the latter distance (Mn–C7 = 2.144(3) Å), as expected for a π-bonding interaction, and the phosphametallacyclic MoPC7C6 ring is essentially planar. The relatively short C6–C7 distance of 1.398(4) Å (cf. 1.399(4) Å in **3a.1**), added to the trigonal environment around these carbon atoms (e.g., Σ(X–C7–Y) = 359.3°), denotes the presence of substantial multiplicity in that bond (1.46 Å expected for a C(*sp*^2^)–C(*sp*^2^) single bond).^15^ In all, the bridging phosphapropenylidene ligand provides the dimetal center with six electrons, which makes the complex electron-precise (34 valence electrons). Therefore, a Mo–Mn single bond has to be formulated for this molecule according to the 18-electron rule, which is consistent with the intermetallic distance of 2.8739(5) Å, noticeably shorter than the analogous distances in the parent compound **1b** (3.1049(3) Å),^7^ and in the unbridged complex [MoMnCp(CO)_8_] (3.083(8) Å),^16^ but still within the wide range of lengths spanned by carbonyl complexes having Mo–Mn single bonds (2.68–3.15 Å).^17^ Even if the coordination mode of the phosphapropenylidene ligands in complexes **2** to **4** is similar to the one observed in the diiron complexes [Fe_2_(μ-κ^2^_P,C_:η^3^-P^*t*^BuCRCH)(CO)_6_] mentioned above,^11^ the interaction of the bridging ligand with the dimetal center is somewhat different in these complexes. In the diiron complexes, two identical Fe(CO)_3_ metal fragments are involved, and the bridging ligand must supply the metal centers with three electrons each. In our complexes, 15-electron (CpMo(CO)_2_) and 13-electron (M(CO)_3_; M = Mn, Re) fragments are involved, so the bridging ligand must provide the metal centers with two and four electrons, respectively. This asymmetric interaction is particularly reflected in the position of the bridging phosphorus atoms, which display relatively large Mo–P separations of ca. 2.55 Å in both **3a.1** and **4b.1**, while distances to the M(CO)_3_ fragments are significantly shorter (2.4538(6) Å for **3a.1** and 2.3011(8) Å for **4b.1**), even after allowing for the respective differences in covalent radii. All of this is consistent with their formulation as P → M donor bonds,^7^ as depicted in Schemes 3 and 4.

**Table 1:** Selected Bond Lengths (Å) and Angles (°) for Compound **4b.1**

Mo1–Mn1	2.8739(5)	Mo1–P1–Mn1	72.61(2)
Mo1–P1	2.541(1)	P1–Mo1–C1	87.6(1)
Mo1–C6	2.167(3)	P1–Mo1–C2	119.7(1)
Mo1–C1	2.032(3)	P1–Mn1–C3	101.2(1)
Mo1–C2	2.004(3)	P1–Mn1–C4	151.8(1)
Mn1–P1	2.3011(8)	P1–Mn1–C5	110.8(1)
Mn1–C7	2.144(3)	C1–Mo1–C2	81.1(1)
Mn1–C6	2.083(3)	C3–Mn1–C4	90.9(2)
Mn1–C3	1.797(3)	C3–Mn1–C5	91.9(2)
Mn1–C4	1.809(3)	C3–Mn1–C7	91.8(1)
Mn1–C5	1.800(3)	C4–Mn1–C5	93.9(2)
P1–C7	1.782(3)	P1–C7–C6	100.0(2)
P1–C15	1.854(3)	P1–C7–C8	134.5(2)
C6–C7	1.398(4)	C6–C7–C8	124.8(3)

Spectroscopic data in solution for compounds **2** to **4** (Table 2 and Experimental section) are similar to each other and are consistent with the solid-state structure discussed above. The IR spectra display in each case four C–O stretching bands for carbonyl ligands, with the high intensity of the most energetic one (at ca. 2020 cm^–1^) denoting the presence of a pyramidal M(CO)_3_ oscillator in these molecules,^18^ while the overall pattern of these bands is comparable to the one observed for the phosphanide- and thiolate-bridged complex [MoReCp(μ-PCy_2_)(μ-SPh)(CO)_5_], as expected.^19^ The formation of the metallacyclic MoPCC ring causes a dramatic shielding of ca. 700 ppm on the ^31^P nuclei of these compounds, relative to the corresponding phosphinidene precursors **1a,b**, to give resonances in the range of −20 to −70 ppm for the rhenium complexes and +10 to +25 ppm for the manganese ones (Table 2). While the shielding of ca. 50 ppm observed when comparing rhenium with manganese products is expected for molecules having phosphanide-like bridging ligands when the metal atoms are replaced with heavier members of the same group,^20^ the absolute values still are significantly lower than expected for complexes having a metal–metal bond (cf. *δ*_P_ + 52.6 ppm in [MoReCp(μ-PCy_2_)(μ-SPh)(CO)_5_]). This additional shielding might be related to the presence of a relatively stressed four-membered MoPCC ring in the molecule.^21^

**Table 2:** Selected IR and ^31^P{^1^H} NMR Data for New Compounds^a^

compound	*ν*(CO)	Δ (P)[^1^*J*_PC_]
[MoReCp(μ-PMes*)(CO)_6_] (**1a**)^b^	2077 (m), 1986 (vs), 1951 (s), 1876 (w)	673.1[28]
[MoMnCp(μ-PMes*)(CO)_6_] (**1b**)^c^	2055 (m), 2039 (w), 1974 (vs), 1951 (s), 1862 (w), 1888 (w)	720.9[30]
[MoReCp{μ-κ^2^_P,C_:η^3^-PMes*C(CO_2_Me)C(CO_2_Me)}(CO)_5_] (**2a.1**)	2027 (vs), 1993 (m), 1948 (m), 1924 (m), 1710 (w)	–30.5
[MoReCp{μ-κ^2^_P,C_:η^3^-PMes*CPhCPh}(CO)_5_] (**2a.2**)	2015 (vs), 1979 (m), 1940 (m), 1908 (m)	–39.3[22]
[MoMnCp{μ-κ^2^_P,C_:η^3^-PMes*C(CO_2_Me)C(CO_2_Me)}(CO)_5_] (**2b.1**)	2025 (vs), 1986 (s), 1953 (m), 1926 (m), 1707 (w)	14.7
[MoReCp{μ-κ^2^_P,C_:η^3^-PMes*CHC(CO_2_Me)}(CO)_5_] (**3a.1**)^d^	2021 (vs), 1985 (m), 1948 (m), 1915 (m), 1691 (w)	–35.9(br)[7]
[MoReCp{μ-κ^2^_P,C_:η^3^-PMes*CHC(^*t*^Bu)}(CO)_5_] (**3a.3**)	2006 (vs), 1966 (m), 1910 (m), 1887 (m)	–70.8[14]
[MoMnCp{μ-κ^2^_P,C_:η^3^-PMes*CHC(CO_2_Me)}(CO)_5_] (**3b.1**)	2019 (vs), 1976 (s), 1951 (m), 1916 (m), 1688 (w)	9.2
[MoReCp{μ-κ^2^_P,C_:η^3^-PMes*C(CO_2_Me)CH}(CO)_5_] (**4a.1**)^d^	2021 (vs), 1987 (m), 1939 (m), 1918 (m), 1733 (w)	–20.5
[MoMnCp{μ-κ^2^_P,C_:η^3^-PMes*C(CO_2_Me)CH}(CO)_5_] (**4b.1**)	2020 (vs), 1980 (s), 1942 (m), 1921 (m), 1697 (w)	26.0(br)[12]
[MoReCp{μ-η^3^:κ^1^_C_-PMes*CHC(CO_2_Me)}(CO)_5_{CN(*p*-C_6_H_4_OMe)}_2_] (**5**)	2177 (w),^e^ 2145 (w),^e^ 2021 (vs), 1973 (m), 1928 (m), 1852 (w)	–21.6[76]
[MoReCp{μ-κ^2^_P,S_:κ ^2^_P,S_-PMes*C(NPh)S}(CO)_5_] (**6**)	2023 (vs), 1989 (m), 1934 (m), 1910 (m)	38.7[10]
[MoReCp(μ-η^2^:κ^1^_S_-SPMes*)(CO)_5_(CNPh)] (**7**)	2156 (w),^e^ 2024 (vs), 1955 (m), 1932 (m), 1911 (m), 1776 (w)	25.4[97]

a IR spectra recorded in dichloromethane solution; ^31^P{^1^H} NMR spectra recorded in CD_2_Cl_2_ solution at 121.48 MHz and 293 K, with chemical shifts (*δ*) in ppm relative to external 85% aqueous H_3_PO_4_, and coupling constants (*J*) in hertz; ^1^*J*_PC_ to the C^1^(C_6_H_2_) carbon, taken from the corresponding ^13^C{^1^H} NMR spectra (see the Experimental section).

b Data taken from ref (5).

c Data taken from ref (7).

d Data taken from ref (6).

e ν(C–N).

Isomers of type **4** give ^31^P resonances somewhat more deshielded than the corresponding isomers of type **3**, but the salient spectroscopic feature enabling the distinction of both groups of compounds is to be found in the P–H coupling of the CH group, which is small (ca. 6 Hz) for isomers of type **3** (mainly a two-bond coupling), and much larger (ca. 46 Hz) for isomers of type **4** (mainly a three-bond coupling, with a H–C–C–P dihedral angle close to 180°).^22^ We note that the diiron complexes [Fe_2_(μ-κ^2^_P,C_:η^3^-P^*t*^BuCRCH)(CO)_6_], which contain phosphapropenylidene ligands analogous to those present in isomers **4**, also displayed very large P-H couplings of ca. 40 Hz.^11^

### Ligand-Induced Rearrangements in Phosphapropenylidene-Bridged Complexes

**Scheme 5:**
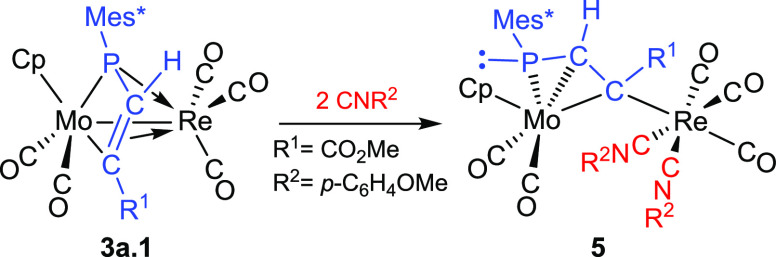
Rearrangement of the Phosphapropenylidene Ligand

As noted above, the formation of compounds **2** to **4** seems to involve first the cycloaddition of an alkyne to the Mo–P double bond of complexes **1**, this being followed by decarbonylation of the M(CO)_4_ fragment and coordination of the C–C double bond of the phosphametallacycle to the resulting M(CO)_3_ fragment (Scheme 4). In order to reverse the last step, and also to check the strength of such a π-bonding coordination, we examined the reaction of a mixture of the MoRe isomers **3a.1** and **4a.1** with CO and with the isocyanide CN(*p*-C_6_H_4_OMe). Carbon monoxide (1 atm) failed to react with the above complexes at room temperature, but the isocyanide reacted smoothly at 273 K with addition of two molecules of ligand to the Re atom, even when using stoichiometric amounts, to give [MoReCp{μ-η^3^:κ^1^_C_-PMes*CHC(CO_2_Me)}(CO)_5_{CN(*p*-C_6_H_4_OMe)}_2_] (**5**), along with unreacted **4a.1** (Scheme 5). We note that complex **5** displays not a phosphametallacyclic structure, but a phosphapropenylidene ligand rearranged into the four-electron donor μ-η^3^:κ^1^_C_ allyl-like coordination mode, with the P atom now bearing a lone electron pair. This rare coordination mode has been previously observed only in the dimolybdenum complexes [Mo_2_Cp_2_(μ-η^3^:κ^1^_C_-PMes*CHCR)(CO)_4_] mentioned above (Scheme 1),^9^ and in the diiron complex [Fe_2_Cp_2_{μ-η^3^:κ^1^_C_-PCyCHC(*p*-tol)}(μ-CO)(CO)], both of them formed under photochemical conditions.^23^ We notice that in all these cases the bridging ligand has a terminal carbon bound to the former phosphinidene ligand. Thus, the reluctance of isomer **4a.1** to undergo a similar rearrangement into the μ-η^3^:κ^1^_C_ mode might be due to steric constrains derived from the proximity of Mes* and CO_2_Me groups in a hypothetical PMes*C(CO_2_Me)CH chain with a P atom also bearing a lone electron pair. In line with this hypothesis, a separate experiment revealed that complex **2a.1** failed to react with CN(*p*-C_6_H_4_OMe) under the same conditions used for **3a.1** (excess of reagent, 273 K).

### Structure of Complex 5

**Figure 2:**
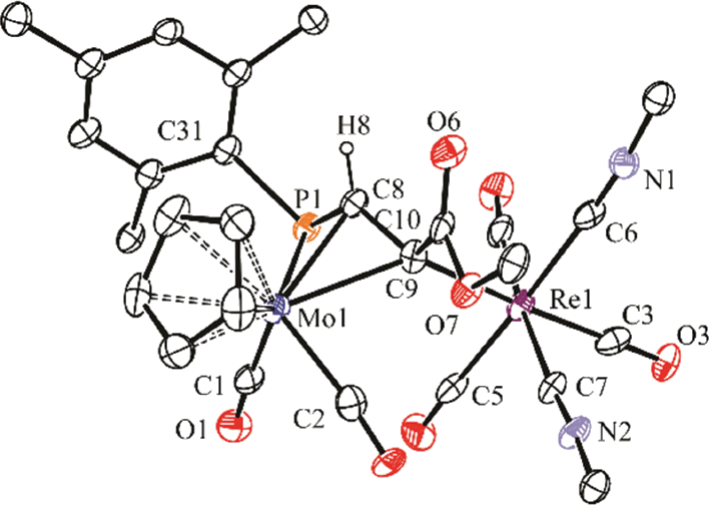
ORTEP diagram (20% probability) of compound **5**, with ^*t*^Bu and *p*-C_6_H_4_OMe groups (except their C^1^ atoms), and most H atoms omitted for clarity.

The molecule of **5** in the crystal (Figure 2 and Table 3) displays a bridging phosphapropenylidene ligand σ-bound to a 17-electron *fac*-Re(CO)_3_(CNR)_2_ fragment (Re–C9 = 2.31(1) Å), thus completing an octahedral environment around the Re atom, while bound to the 15-electron CpMo(CO)_2_ fragment in an η^3^, allyl-like fashion, with an *exo* conformation of the PCC chain (P1–C8–C9 = 120.6(8)°) relative to the metal fragment.^24^ The relatively short C8–C9 distance of 1.41(1) Å is similar to the ones measured in the parent complex **3a.1** and its manganese analogue **4b.1** (ca. 1.40 Å), therefore indicative of the presence of substantial multiplicity in that bond, and the same can be said of the P–C8 distance (1.75(1) Å), significantly below the reference value of 1.80 Å for a P–C(sp^2^) bond,^15^ and somewhat shorter than the corresponding distance in the parent complex **3a.1** (1.770(3) Å).^6^ The external carbon atom of the chain displays a Mo–C separation significantly longer than the internal carbon (Mo–C9 = 2.40(1) vs Mo–C8 = 2.26(1) Å), as expected. Besides this, the Mo–P distance of 2.653(3) Å is quite large too, as anticipated for a π- (instead of σ-) bonding interaction, and comparable to the distance of 2.6646(7) Å measured in the related homometallic complex [Mo_2_Cp_2_{μ-η^3^:κ^1^_C_-PMes*CHC(*p*-tol)}(CO)_4_]. For comparison with conventional σ-bond lengths, we note that the reference figure for a Mo–P single bond is 2.61 Å,^15^ while the W–P distance in the pyramidal phosphanide complex [WCp(CO)_3_(PC_4_H_2_Me_2_)] is 2.571(2) Å.^25^ In any case, the 4-electron contribution of the bridging organophosphorus ligand to the dimetal center makes compound **5** a 36-electron complex, for which no metal–metal bond has to be proposed according to the 18-electron rule. This is in agreement with the very large intermetallic separation of 4.270(1) Å, which precludes the concurrence of any bonding interaction between the metal centers.

**Table 3:** Selected Bond Lengths (Å) and Angles (°) for Compound **5**

Mo1····Re1	4.270(1)	Mo1–C9–Re1	130.3(5)
Mo1–P1	2.653(3)	P1–Mo1–C1	73.7(3)
Mo1–C8	2.26(1)	P1–Mo1–C2	114.5(4)
Mo1–C9	2.40(1)	P1–C8–C9	120.6(8)
Mo1–C1	1.95(1)	C9–Re1–C3	175.6(4)
Mo1–C2	1.96(1)	C9–Re1–C4	93.8(4)
Re1–C9	2.31(1)	C9–Re1–C5	93.0(4)
Re1–C3	1.94(1)	C9–Re1–C6	84.2(4)
Re1–C4	2.00(2)	C9–Re1–C7	89.0(4)
Re1–C5	2.03(1)	C4–Re1–C7	176.3(4)
Re1–C6	2.10(2)	C5–Re1–C6	177.2(4)
Re1–C7	2.11(1)	C6–Re1–C7	88.3(4)
P1–C8	1.75(1)	C1–Mo1–C2	78.7(5)
P1–C31	1.88(1)		
C8–C9	1.41(1)		

Spectroscopic data in solution for compound **5** (Table 2 and Experimental Section) are consistent with the solid-state structure of the complex. Its IR spectrum displays two C–N and four C–O stretches that can be considered as arising from essentially independent Mo(CO)_2_ and Re(CO)_3_(CNR)_2_ oscillators, an assumption consistent with the absence of a metal–metal bond connecting them. The C–N stretches of the isocyanide ligands are of similar intensity, which is indicative of their cisoid arrangement (cf. C6–Re–C7 = 88.3(4)° in the crystal), while the high intensity of the most energetic C–O stretch (2021 cm^–1^) denotes the facial arrangement of the three carbonyl ligands bound to Re.^18^ Compound **5** displays a ^31^P NMR resonance at −21.6 ppm, only some 15 ppm above that of the parent compound **3a.1**, not an unusual position in this case for a lone pair-bearing P atom (cf. *δ*_P_ −31.9 ppm for the pyramidal phosphanide complex [MoCp(PPh_2_)(CO)_2_(PMe_3_)]),^26^ and the ^1^H and ^13^C NMR resonances of the atoms of the P–C–C chain are comparable to those previously measured for the dimolybdenum complexes [Mo_2_Cp_2_(μ-η^3^:κ^1^_C_-PMes*CHCR)(CO)_4_] (R = *p*-tol, CO_2_Me, ^*i*^Pr), then deserving no detailed comments. We note however that the aryl carbon bound to phosphorus in **5** displays an unusually large one-bond P–C coupling of 76 Hz, comparable to the values of ca. 80 Hz measured in the above Mo_2_ complexes. This is a spectroscopic feature that seems to be related to the presence of a lone pair at the P atom, also found in κ^1^_S_:η^2^-bridged thiophosphinidene complexes, as discussed below. A similar effect applies to the P-bound CH carbon of the PCC chain, which displays an unusually large P–C coupling of 54 Hz (cf. 11 Hz in the precursor **3a.1**).

### Reactions of Compounds **1** with Phenyl Isothiocyanate

**Scheme 6:**
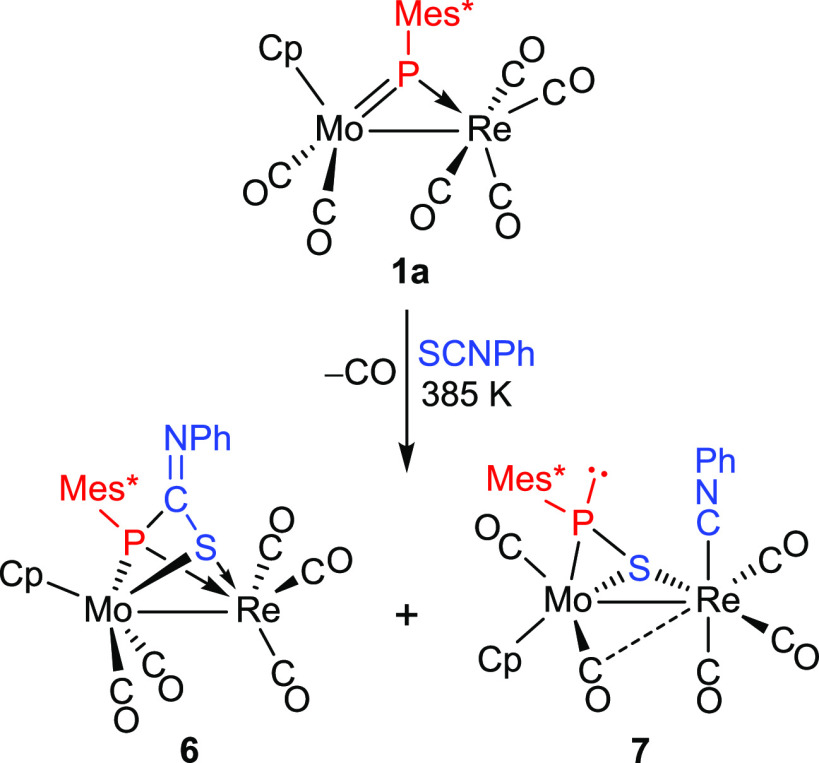
Reaction of Compound 1a with Phenyl Isothiocyanate

The rhenium complex **1a** failed to react with a slight excess of phenyl isothiocyanate at room temperature but did react slowly at 363 K in toluene solution, or more rapidly under refluxing conditions, to give as major products the phosphametallacyclic complex [MoReCp{μ-κ^2^_P,S_:κ^2^_P,S_-PMes*C(NPh)S}(CO)_5_] (**6**), and its thiophosphinidene-bridged isomer [MoReCp(μ-η^2^:κ^1^_S_-SPMes*)(CO)_5_(CNPh)] (**7**), which were isolated in ca. 20 and 30% yields respectively (Scheme 6). Small amounts of the known hydride [MoReCp(μ-H){μ-P(CH_2_CMe_2_)C_6_H_2_^*t*^Bu_2_}(CO)_6_] (an isomer of the parent complex **1a**),^6^ and other minor uncharacterized species were also formed in this reaction. The manganese complex **1b** reacted with phenyl isothiocyanate under similar conditions, but an even more complex mixture of products were obtained, which were not further investigated.

Compound **6** formally follows from the expected [2 + 2] cyloaddition between C=S and Mo=P bonds, with specific formation of a P—C bond, to render a MoPCS phosphametallacycle, as observed in the Mo_2_ complex depicted in Scheme 2. However, decarbonylation at the Re(CO)_4_ fragment now follows, this inducing further coordination of the S atom to the Re center, to render a *P*- and *S*-bridging ligand equivalent to independent phosphanide and thiolate ligands, as those present in complex [MoReCp(μ-PCy_2_)(μ-SPh)(CO)_5_].^19^ We note that this six-electron donor coordination mode of a phosphinidene-isothiocyanate adduct (μ-κ^2^_P,S_:κ^2^_P,S_) has not been structurally characterized previously.

Complex **7** is an isomer of **6** but instead displays a terminal isocyanide ligand bound to the Re atom and a thiophosphinidene ligand bridging the metal atoms in the μ-κ^1^_S_:η^2^ coordination mode. The latter is an unusual coordination mode for a bridging thiophosphinidene ligand, which usually adopts the μ-κ^1^_P_:η^2^ coordination mode, as invariably found upon reactions of trigonal phosphinidene-bridged complexes of types **B** and **C** with sulfur.^27−29^ Actually, the μ-κ^1^_S_:η^2^ coordination mode has been identified for the first time only recently, in the related carbonyl-only containing complex [MoReCp(μ-η^2^:κ^1^_S_-SPMes*)(CO)_6_] (**8**), a product formed slowly in the reaction of **1a** with sulfur in toluene solution at moderate temperature (313 K).^30^ It is interesting to note that the latter complex underwent fast decarbonylation at 363 K in toluene solution, this inducing rearrangement of its thiophosphinidene ligand into the six-electron donor μ-η^2^:η^2^ coordination mode. Such a rearrangement would be less favored in the case of **7**, as a Re(CO)_3_(CNR) fragment is expected to be more reluctant than a Re(CO)_4_ one to undergo decarbonylation; this would enable the persistence of **7** even in boiling toluene solutions (ca. 385 K).

**Scheme 7:**
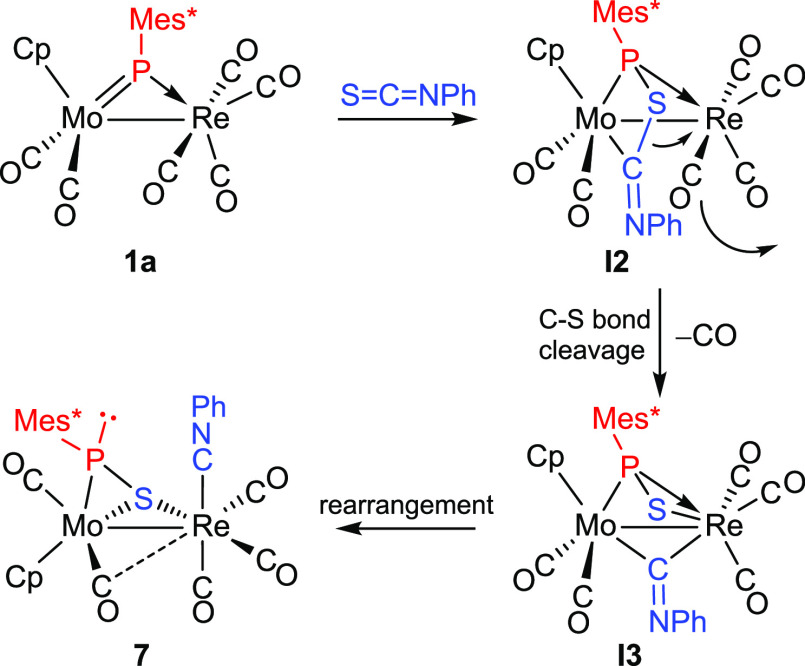
Proposed Reaction Path to Compound **7**

Since **7** and **6** are isomeric species, a natural question to ask is whether **7** might be possibly formed from **6**. However, this does not seem to be the case, as a separated experiment indicated that a toluene solution of **6** underwent no detectable transformations under refluxing conditions after 3 h. Instead, we propose that **7** is derived from an alternative cycloaddition of Mo=P and C=S bonds, now involving formation of P–S and Mo–C bonds to give an intermediate **I2** with a MoPSC (instead of MoPCS) phosphametallacycle (Scheme 7). Decarbonylation of the Re fragment now might trigger the oxidative addition of the S–C bond of the cycle to give a second intermediate **I3** with bridging isocyanide and thiophosphinidene ligands. The coordination of the SPMes* ligand stemming from that step would be one of the more common μ-κ^1^_P_:η^2^ type, it rearranging rapidly into the less usual μ-κ^1^_S_:η^2^ mode, more favored thermodynamically in this case, as shown by theoretical calculations to be discussed later on. This would be accompanied by a conventional rearrangement of the isocyanide ligand, from bridging to terminal coordination at the Re center, to eventually render the isolable product **7**. The fact that **7** is formed in higher amounts than **6** (ca. 3 to 2 ratio) would be explained by taking into account that, in the initial cycloaddition step, the formation of a P–S bond would be favored over the P–C one on steric grounds, as in this way the bulkier part of the heterocumulene (the NPh group) is placed away from the bulky Mes* group. Steric effects thus would operate in the same way observed for the reactions of compounds **1** with terminal alkynes.

### Structure of Compound **6**

**Figure 3:**
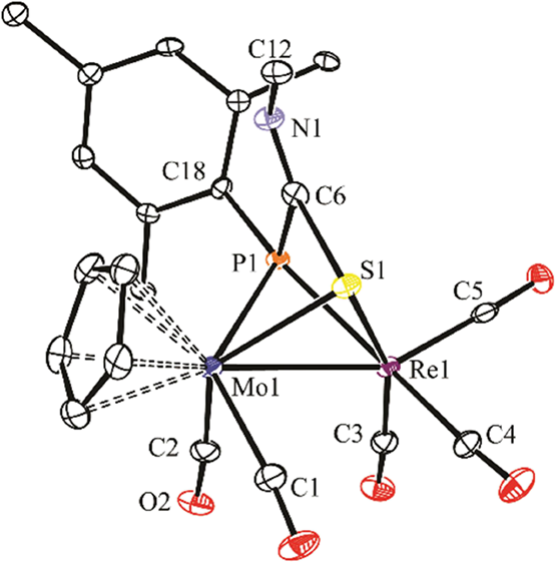
ORTEP diagram (30% probability) of compound **6**, with ^*t*^Bu and Ph groups (except their C^1^ atoms) and H atoms omitted for clarity.

The molecule of this complex in the crystal (Figure 3 and Table 4) displays MoCp(CO)_2_ and pyramidal Re(CO)_3_ fragments bridged through the P and S atoms of the phosphinidene-isothiocyanate adduct RPC(NPh)S in a novel μ-κ^2^_P,S_:κ^2^_P,S_ fashion, with the central C=NPh moiety (C–N = 1.244(7) Å) not interacting with the metal atoms. The ligand thus configures a geometric environment equivalent to phosphanide- and thiolate-bridging ligands, as found in the mentioned complex [MoReCp(μ-PCy_2_)(μ-SPh)(CO)_5_]. In order to balance the distinct electron counts of the Mo and Re fragments (15 and 13 electrons respectively), the bridgehead P and S atoms are expected to bind more tightly the Re atom, although alternative ways are possible (e.g., formation of a dative Mo → Re bond). The former seems to be the case of phosphorus (Mo–P = 2.576(1) vs Re–P = 2.436(1) Å), but coordination of the S atom can be classified as symmetrical (Mo–S = 2.490(1) vs Re–S 2.461(1) Å), after accounting for the ca. 0.03 Å difference in the covalent radii of the metal atoms. In any case, the bridging ligand provides the dimetal center with six electrons, thus rendering a 34-electron complex for which a Mo–Re single bond has to be proposed according to the 18-electron rule. This is consistent with the observed intermetallic length of 2.9757(4) Å, almost identical to the value of 2.9702(8) Å measured in the mentioned thiolate complex.^19^ The endocyclic P–C6 distance of 1.825(5) Å is comparable to the one measured in the only other phosphinidene-thiocyanate complex structurally characterized to date (the Mo_2_ complex depicted in Scheme 2) and compares well with the reference value for a P–C(sp^2^) single bond (1.80 Å), but the S–C6 distance of 1.849(5) Å is longer than expected, with no obvious reason for it. The corresponding distance in the mentioned Mo_2_ complex is 1.766(3) Å,^13^ and those in related MPR_2_C(NR)S metallacycles similarly approach the reference figure of ca. 1.78 Å for a S–C(sp^2^) single bond.^31,32^

**Table 4:** Selected Bond Lengths (Å) and Angles (°) for Compound **6**

Mo1–Re1	2.9757(4)	Mo1–P1–Re1	72.78(3)
Mo1–P1	2.576(1)	Mo1–S1–Re1	73.87(3)
Mo1–S1	2.490(1)	P1–Mo1–C1	127.0(2)
Mo1–C1	2.018(6)	P1–Mo1–C2	88.0(2)
Mo1–C2	1.981(6)	P1–Re1–C3	106.6(2)
Re1–P1	2.436(1)	P1–Re1–C4	158.0(2)
Re1–S1	2.461(1)	P1–Re1–C5	101.0(2)
Re1–C3	1.908(6)	C1–Mo1–C2	78.0(2)
Re1–C4	1.943(5)	C3–Re1–C4	92.7(2)
Re1–C5	1.900(6)	C3–Re1–C5	89.6(2)
P1–C6	1.825(5)	P1–C6–S1	96.0(2)
S1–C6	1.849(5)	P1–C6–N1	131.6(4)
C6–N1	1.244(7)	S1–C6–N1	131.3(4)
P1–C18	1.843(4)	C6–N1–C12	127.0(4)

Spectroscopic data in solution for compound **6** (Table 2 and Experimental section) are consistent with the solid-state structure of the complex. Its IR spectrum displays four C–O stretches for the carbonyl ligands, with a pattern comparable to that of the phosphapropenylidene-bridged compounds **2** to **4**, as expected when considering the similarly arranged Mo(CO)_2_ and M(CO)_3_ oscillators, and the ^1^H and ^13^C NMR resonances for the bridging ligand are not very different from those of the mentioned Mo_2_ complex, then deserving no particular comments. The bridgehead P atom in **6** gives rise to a NMR resonance at 38.7 ppm, a chemical shift lower than the one measured in the phosphanide-bridged complex [MoReCp(μ-PCy_2_)(μ-SPh)(CO)_5_] (*δ*_P_ 52.6 ppm), a shielding effect perhaps related to the presence of 4-membered phosphametallacycles in **6**.^21^

### Structure of Compound **7**

**Figure 4:**
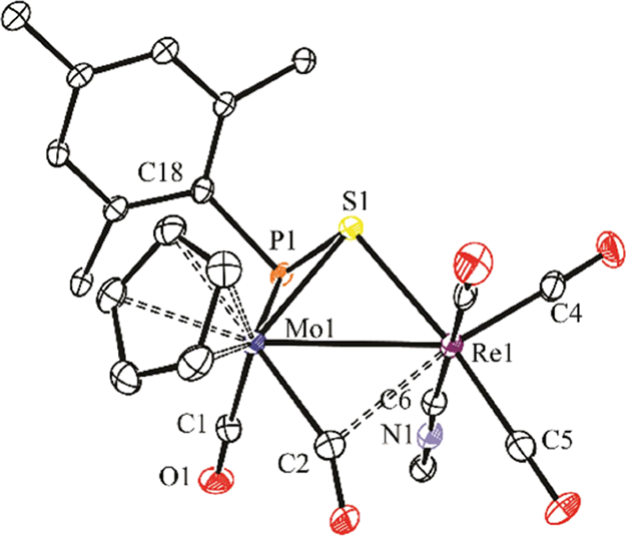
ORTEP diagram (30% probability) of compound **7**, with ^*t*^Bu and Ph groups (except their C^1^ atoms) and H atoms omitted for clarity.

The molecule of this complex in the crystal (Figure 4 and Table 5) is very similar to the one recently determined for its hexacarbonyl analogue **8**,^30^ except for the presence of the terminal isocyanide ligand bound to the Re atom instead of a carbonyl and placed *cis* to the bridging S atom (S–Re–C6 = 94.1(2)°), thus leaving the carbonyl ligands in a relative facial arrangement. The intermetallic distance, the presence of a semibridging carbonyl ligand (Re···C2 = 2.499(6) Å), and the geometrical parameters of the bridging thiophosphinidene ligand are all comparable to those determined for compound **8** and deserve no additional comments.

**Table 5:** Selected Bond Lengths (Å) and Angles (°) for Compound **7**

Mo1–Re1	3.0358(4)	Mo1–S1–Re1	77.45(4)
Mo1–P1	2.642(1)	P1–Mo1–C1	71.8(2)
Mo1–S1	2.425(1)	P1–Mo1–C2	116.6(2)
Mo1–C1	1.961(6)	S1–Re1–C3	87.6(2)
Mo1–C2	1.991(6)	S1–Re1–C4	96.7(2)
Re1–S1	2.428(1)	S1–Re1–C5	171.6(2)
Re1–C3	1.969(6)	S1–Re1–C6	94.1(2)
Re1–C4	1.909(6)	C1–Mo1–C2	76.1(2)
Re1–C5	1.928(7)	C3–Re1–C4	91.3(2)
Re1–C6	2.080(6)	C3–Re1–C5	88.8(2)
Re1···C2	2.499(6)	C3–Re1–C6	175.9(2)
P1–S1	2.090(2)	C4–Re1–C5	91.0(3)
P1–C18	1.863(5)	Mo1–C2–O2	155.1(5)
C6–N1	1.159(8)	C6–N1–C7	175.4(6)

The IR and NMR data in solution for **7** (Table 1 and Experimental section) are consistent with the solid-state structure of the molecule and are also comparable to those of the all-carbonyl complex **8**, except for features derived from the presence of the isocyanide ligand. The latter gives rise to a weak C–N stretch at 2156 cm^–1^ in the IR spectrum, whereas the Re(CO)_3_ oscillator gives rise to three C–O stretches, the most energetic one being of high intensity, as expected from the facial arrangement of the carbonyls found in the crystal. At the same time, the presence of a relatively red-shifted C–O stretch at 1776 cm^–1^ is indicative of the retention of the semibridging carbonyl in solution, identified as a Mo-bound carbonyl on the basis of its significantly deshielded resonance at 242.2 ppm in the ^13^C NMR spectrum (cf. 232.1 ppm for the terminal Mo–CO). This spectrum also reveals an unusually large one-bond P-C coupling of 97 Hz for the *ipso* carbon in the aryl group of the thiophosphinidene ligand, identical to the one found for **8**. This very large P–C coupling, also found in complex **5** and related homometallic Mo_2_ complexes,^9^ seem to be associated with the presence of a lone electron pair at the P atom^30^ and therefore seems to be of use as a reliable spectroscopic signature to detect such an electronic feature in new compounds to be made in future.

### Isomerism in Thiophosphinidene-Bridged Complexes: κ^1^_P_:η^2^ vs κ^1^_S_:η^2^ Coordination Modes

**Figure 5:**
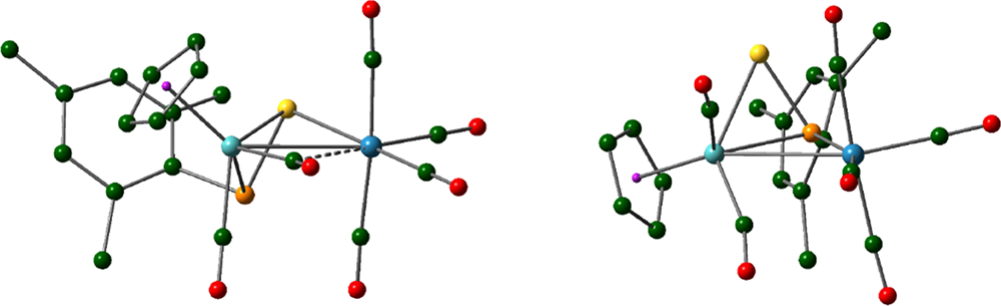
M06L-DFT-computed structures of isomers **8** (left) and **8-P** (right), with ^*t*^Bu groups (except their C^1^ atoms) and H atoms omitted for clarity.

We were intrigued by the fact that all previous κ^1^:η^2^-bridged thiophosphinidene complexes structurally characterized so far display their P atoms at the bridgehead position (κ^1^_P_:η^2^ mode), while both complex **7** and its carbonyl-only analogue **8** display bridging S atoms instead (κ^1^_S_:η^2^ mode). At first glance, we could not know whether this might follow from a thermodynamic or kinetic preference. A significant difference between these two groups of complexes is that the former group includes complexes with metal fragments positioned rather far away from each other, because of the absence of a metal–metal bond in them, whereas complexes **7** and **8** are metal–metal bound complexes with metal fragments much closer to each other (Mo–Re ca. 3.05 Å). This might lead to significant differences in the steric crowding of these two groups of complexes. To gain further insight into this matter, we have performed density functional theory (DFT) calculations on **7** and **8** with the thiophosphinidene ligands both in their experimentally determined geometries (κ^1^_S_:η^2^ mode) and in their κ^1^_P_:η^2^ isomeric forms. The optimized geometries for **8** and its κ^1^_P_:η^2^-bridged isomer **8-P** are shown in Figure 5, while the ones for **7** and their two possible κ^1^_P_:η^2^-bridged isomers (***syn*-7P** and ***anti*-7P**, differing in the positioning of the CNPh ligand relative to the S atom) are shown in Figure S37. Table 6 collects the most relevant geometrical parameters involving the bridging ligands, as well as the relative energies of the computed species.

**Table 6:** Selected M06L-DFT-Computed Bond Lengths (Å) for Complexes **7** to **9** and Their Isomers^a^

Param	**7**	***syn*-7P**	***anti*-7P**	**8**	**TS1**	**8-P**	**9**	**TS2**	**9-P**
Mo–Re	3.084	3.160	3.223	3.068	3.155	3.168			
Mo–P	2.661	2.498	2.556	2.670	2.582	2.489	2.620	2.540	2.537
Mo–S	2.461	2.585	2.572	2.457	2.495	2.562	2.493	2.510	2.536
Re–S	2.510			2.500	3.065		2.611	3.160	
Re–P		2.473	2.447		2.860	2.468		3.097	2.671
P–S	2.116	2.044	2.053	2.118	2.096	2.061	2.127	2.104	2.053
Δ*G*	0	+77	+61	0	+91	+29	0	+55	–6

a Relative Gibbs free energies at 298.15 K (Δ*G*) given in kJ/mol as the last entry, relative to the κ^1^_S_:η^2^ isomers. The central MoPSRe core for different isomers of compounds **7** to **9** is sketched above, with L = CNPh, *Mo* = MoCp(CO)_2_ in all cases, and *Re* = Re(CO)_3_ (**7**), Re(CO)_4_ (**8**) and Re(CO)_5_ (**9**).
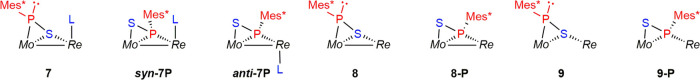

First, we note the good agreement between the experimental and computed structural parameters for both **7** and **8**. As it can be appreciated from the data in this table, the interatomic distances involving the bridging ligand are not modified significantly when replacing the isocyanide with a carbonyl ligand (**7** vs **8**), nor they are the intermetallic separations. Noticeably, the computed structures for the κ^1^_S_:η^2^-SPMes*-bridged isomers (**7** and **8**) are the ones with the lower Gibbs free energies in each case, whereas the corresponding κ^1^_P_:η^2^-bridged isomers (***syn*-** and ***anti*-7P** or **8-P**) are some 30 to 80 kJ/mol less stable, so there is no doubt that the κ^1^_S_:η^2^-bridged isomers are the thermodynamically preferred isomers in these metal–metal bound thiophosphinidene complexes. Interestingly, the κ^1^_S_:η^2^ isomers display intermetallic distances ca. 0.1 Å shorter than their κ^1^_P_:η^2^ counterparts, and we take this as an indication that the steric pressure at the dimetal center is somewhat lower in the former isomers, as suspected, this allowing for a closer approach of the metal fragments, therefore for better orbital overlap and eventually to an increased thermodynamic stability. In line with this, the energetic difference for the isomers of compound **7** (ca. 60 to 80 kJ/mol) is higher than the one for compound **8** (29 kJ/mol), the latter bearing a sterically less demanding Re(CO)_4_ fragment (vs Re(CO)_3_(CNPh)).

Compound **8** is the first product detected in the reaction of the phosphinidene complex **1a** with elemental sulfur.^30^ Since the reactivity of **1a** seems to be dominated by the cycloaddition reactions at the Mo=P double bond of this molecule, it would be sensible to hypothesize that reaction of **1a** with elemental sulfur would lead initially to isomer **8-P**, following from a [2 + 1] cycloaddition of an S atom to the Mo=P double bond of the molecule, as observed in the reactions of phosphinidene complexes of types **B** and **C** (Chart 1) with sulfur.^27−29^ Since **8-P** is less stable than the actual product of the above reaction (**8**), then the natural question to answer is whether isomerization from **8-P** to **8** would be a kinetically accessible process. DFT calculations in search for a transition state connecting both isomers indeed located such a structure (**TS1**), it being only some 62 kJ/mol higher in energy than **8-P**, therefore allowing for a very fast rearrangement of the latter into the most stable isomer **8** at room temperature. Interestingly, **TS1** displays a thiophosphinidene ligand with the P atom significantly detached from the Re center (Re···P = 2.860 Å) while the S atom still is at an almost nonbonding distance of the latter (Re···S = 3.065 Å). Thus, it might seem that the intermetallic bond, which is being shortened along the process (Mo–Re = 3.155 Å at **TS1**), is a significant support of the nuclearity of the complex at this transient stage.

**Chart 2:**
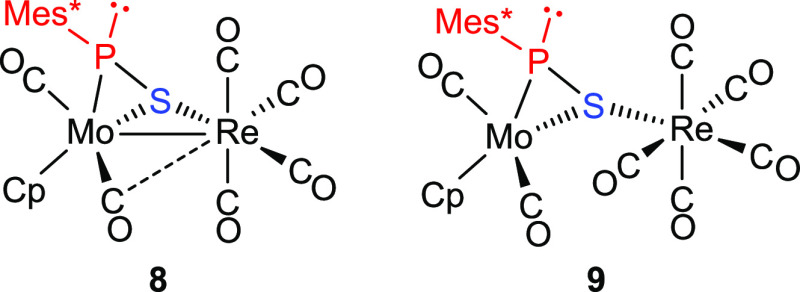
Carbonyl-Only Complexes Related to **7**

To gain further knowledge on the influence of the intermetallic bond in the above isomerization, we have also computed the structures of the hypothetical heptacarbonyl complex **9** (Chart 2) and that of its κ^1^_P_:η^2^-bridged isomer (**9-P**). Because of the extra carbonyl in **9** (vs **8**), no intermetallic bond is to be present in this molecule according to the 18-electron rule, which is consistent with the large intermetallic separation computed for both isomers of this complex (above 4.4 Å, see the SI). Interestingly, the κ^1^_S_:η^2^-bridged isomer **9** is now computed to be slightly less stable (by 6 kJ/mol) than the κ^1^_P_:η^2^ one (**9-P**). In any case, the rearrangement between **9** and **9-P** is also expected to be very fast at room temperature, since the corresponding transition state (**TS2**), with a coordination of the SPMes* ligand similar to the one found in **TS1**, has an energy only 62 kJ/mol above the most stable isomer **9-P**. So in all we must conclude that the presence or absence of an intermetallic interaction itself is of not much relevance to determine the kinetic barrier for the rearrangement of κ^1^_P_:η^2^ into κ^1^_S_:η^2^ isomers of these thiophosphinidene-bridged complexes, which is expected to be generally fast at room temperature. However, the thermodynamic stability of the κ^1^_P_:η^2^ isomers is significantly reduced for the metal–metal bound complexes, due to increased steric crowding derived from the closer mutual approach of the metal fragments, thus making the κ^1^_S_:η^2^ isomers thermodynamically preferred in these cases.

## Conclusions

The reactions of complexes **1a,b** with alkynes R^1^C≡CR^2^ (R^1^ = R^2^, H) are apparently initiated with a [2 + 2] cycloaddition step between Mo=P and C≡C bonds that builds MoPCC metallacycles, with preferential P–CH coupling in its case, to minimize steric repulsions. This would be followed by easy decarbonylation at the M(CO)_4_ fragment, which induces coordination of the C=C double bond of the metallacycle to eventually give phosphapropenylidene-bridged complexes of type [MoMCp(μ-κ^2^_P,C_:η^3^-PMes*CR^1^CR^2^)(CO)_5_] [R^1^ = R^2^ (**2**); R^1^ = H (**3**); R^2^ = H (**4**)]. Addition of ligands promotes rearrangement of the bridging organophosphorus ligand into the rare, allyl-like μ-η^3^:κ^1^_C_ coordination mode with no need of photochemical activation, but only when R^1^ = H, as observed in the reaction of [MoReCp(μ-κ^2^_P,C_:η^3^-PMes*CHC(CO_2_Me)}(CO)_5_] with CN(*p*-C_6_H_4_OMe) to give [MoReCp{μ-η^3^:κ^1^_C_-PMes*CHC(CO_2_Me)}(CO)_5_{CN(*p*-C_6_H_4_OMe)}_2_] (**5**). Presumably, steric constrains within an hypothetical PMes*CR^1^CR^2^ chain having a lone pair-bearing P atom and R^1^ other than H precludes formation of similar allyl-like complexes. Complex **1a** also undergoes cycloaddition reactions with S=C=NPh, giving as major products the phosphametallacyclic complex [MoReCp{μ-κ^2^_P,S_:κ^2^_P,S_-PMes*C(*NPh*)S}(CO)_5_] (**6**), and its thiophosphinidene-bridged isomer [MoReCp(μ-η^2^:κ^1^_S_-SPMes*)(CO)_5_(CNPh)] (**7**). Compound **6** seems to follow from a [2 + 2] cycloaddition between Mo=P and C=S bonds, with specific P–C coupling, whereas **7** would arise from the alternative cycloaddition, more favored on steric grounds, involving P–S coupling. Decarbonylation of the Re(CO)_4_ in the first case would promote rearrangement of the bridging ligand into the novel μ-κ^2^_P,S_:κ^2^_P,S_ coordination mode, while in the second case would induce C–S bond cleavage to yield isocyanide and thiophosphinidene ligands. The prevalence of the rare μ-κ^1^_S_:η^2^ coordination mode of the thiophosphinidene ligand in **7** over the more common μ-κ^1^_P_:η^2^ mode and the interconversion between both coordination modes were investigated using DFT calculations on **7** and related carbonyl-only analogues. It is concluded that the presence or absence of an intermetallic interaction itself has little influence on the kinetic barrier for the rearrangement of κ^1^_P_:η^2^ into κ^1^_S_:η^2^ isomers of these thiophosphinidene complexes, which remains low in all cases. However, the thermodynamic stability of the κ^1^_P_:η^2^ isomers is significantly reduced for the metal–metal bound complexes, due to increased steric repulsions between metal fragments, thus making the κ^1^_S_:η^2^ isomers thermodynamically preferred in these cases.

## Experimental Section

### General Procedures and Starting Materials

All manipulations and reactions were carried out under an argon (99.995%) atmosphere using standard Schlenk techniques. Solvents were purified according to literature procedures and distilled prior to use.^33^ Compounds [MoReCp(μ-PMes*)(CO)_6_] (**1a**),^6^ and [MoMnCp(μ-PMes*)(CO)_6_] (**1b**),^7^ were prepared as described previously (Cp = η^5^-C_5_H_5_; Mes* = 2,4,6-C_6_H_2_^*t*^Bu_3_). The preparation of compounds **3a.1** and **4a.1** was described in our preliminary report on this chemistry.^6^ All other compounds were obtained from commercial suppliers and used as received, unless otherwise stated. Petroleum ether refers to that fraction distilling in the range of 338–343 K. Filtrations were carried out through diatomaceous earth unless otherwise stated. Chromatographic separations were carried out using jacketed columns cooled by a closed 2-propanol circuit kept at 258 K with a cryostat. Commercial aluminum oxide (activity I, 70–290 mesh) was degassed under vacuum prior to use. The latter was mixed under argon with an appropriate amount of water to reach activity IV. IR stretching frequencies of CO ligands were measured in solution (using CaF_2_ windows), are referred to as ν(CO), and are given in wave numbers (cm^–1^). Nuclear magnetic resonance (NMR) spectra were routinely recorded at 295 K unless otherwise stated. Chemical shifts (*δ*) are given in ppm, relative to internal tetramethylsilane (^1^H, ^13^C), or external 85% aqueous H_3_PO_4_ solutions (^31^P). Coupling constants (*J*) are given in hertz.

### Preparation of [MoReCp{μ-κ^2^_P,C_:η^3^-PMes*C(CO_2_Me)C(CO_2_Me)}(CO)_5_] (**2a.1**)

Dimethyl acetylenedicarboxylate (10 μL, 0.081 mmol) was added to a toluene solution (8 mL) of compound **1a** (0.020 g, 0.025 mmol), and the mixture was stirred at 333 K for 3 h to give an orange-brown solution. After removal of the solvent under vacuum, the residue was extracted with dichloromethane/petroleum ether (1/8), and the extracts were chromatographed on alumina at 258 K. Elution with the same solvent mixture gave minor gray and yellow fractions containing small amounts of unidentified species. Elution with dichloromethane/petroleum ether (1/4) gave a yellow fraction yielding, upon removal of solvents, compound **2a.1** as a yellow microcrystalline solid (0.018 g, 78%). Anal. Calcd for C_34_H_40_MoO_9_PRe: C, 45.08; H, 4.45. Found: C, 44.83; H, 4.05. ^1^H NMR (400.13 MHz, CD_2_Cl_2_): *δ* 7.31, 7.24 (2s, 2 × 1H, C_6_H_2_), 5.65 (s, 5H, Cp), 3.62, 3.12 (2s, 2 × 3H, OMe), 1.57 (s, br, 18H, *o*-^*t*^Bu), 1.32 (s, 9H, *p*-^*t*^Bu). ^13^C{^1^H} NMR (100.63 MHz, CD_2_Cl_2_): *δ* 229.5 (d, *J*_CP_ = 9, MoCO), 229.3 (d, *J*_CP_ = 13, MoCO), 196.3 (s, br, 3ReCO), 172.9 (d, *J*_CP_ = 25, *C*O_2_Me), 163.9 (d, *J*_CP_ = 3, *C*O_2_Me), 161.6 [s, br, C^1^(C_6_H_2_)], 158.6 [s, br, C^4^(C_6_H_2_)], 152.2 [d, *J*_CP_ = 4, C^2,6^(C_6_H_2_)], 128.7 [s, C^6,2^(C_6_H_2_)], 123.8 [d, *J*_CP_ = 11, C^3,5^(C_6_H_2_)], 122.3 [d, *J*_CP_ = 8, C^5,3^(C_6_H_2_)], 118.8 (s, br, *C*CO_2_Me), 113.3 (d, *J*_CP_ = 6, *C*CO_2_Me), 92.2 (s, Cp), 52.6, 51.7 (2s, OMe), 40.0, 39.5 [2s, br, C^1^(*o*-^*t*^Bu)], 35.1 [s, C^1^(*p*-^*t*^Bu)], 34.5, 33.2 [2s, br, C^2^(*o*-^*t*^Bu)], 31.3 [s, C^2^*(p*-^*t*^Bu)].

### Preparation of [MoReCp{μ-κ^2^_P,C_:η^3^-PMes*CPhCPh}(CO)_5_] (**2a.2**)

Diphenylacetylene (0.010 g, 0.056 mmol) was added to a toluene solution (8 mL) of compound **1a** (0.020 g, 0.025 mmol), and the mixture was stirred at 333 K for 12 h to give an orange-brown solution. Workup was similar to the one described for **2a.1**. Elution with dichloromethane/petroleum ether (1/8) gave first a minor fraction of unreacted **1a**, then a yellow fraction yielding compound **2a.2** as a yellow microcrystalline solid (0.018 g, 75%). Anal. Calcd for C_42_H_44_MoO_5_PRe: C, 53.56; H, 4.71. Found: C, 53.58; H, 4.78. ^1^H NMR (400.13 MHz, CD_2_Cl_2_): *δ* 7.41, 6.94 (2s, br, 2 × 1H, C_6_H_2_), 7.07–6.99 (m, 3H, Ph), 6.89–6.83 (m, 3H, Ph), 6.70 (t, *J*_HH_ = 8, 2H, Ph), 6.02 (d, *J*_HH_ = 8, 2H, Ph), 5.66 (s, 5H, Cp), 1.66, 1.38 (2s, br, 2 × 9H, *o*-^*t*^Bu) 1.33 (s, 9H, *p*-^*t*^Bu). ^13^C{^1^H} NMR (100.63 MHz, CD_2_Cl_2_): *δ* 232.7 (s, br, MoCO), 232.4 (d, *J*_CP_ = 13, MoCO), 197.9 (d, *J*_CP_ = 7, 3ReCO), 162.9, 158.4 [2s, br, C^2,6^(C_6_H_2_)], 152.5 [d, *J*_CP_ = 4, C^4^(C_6_H_2_)], 148.6 [d, *J*_CP_ = 22, C^1^(C_6_H_2_)], 138.5 (s, *C*Ph), 138.1, 135.0 [2s, C^1^(Ph)], 131.0 [s, C^2^(Ph)], 130.7 [d, *J*_CP_ = 3, C^2^(Ph)], 127.5 [s, C^3^(Ph)], 127.0, [s, C^4^(Ph)], 126.8 [s, C^3^(Ph)], 126.1 [s, C^4^(Ph)], 124.1, 122.5 [2s, C^3,5^(C_6_H_2_)], 118.1 (s, *C*Ph), 92.3 (s, Cp), 40.3, 39.6 [2s, br, C^1^(*o*-^*t*^Bu)], 35.1 [s, C^1^(*p*-^*t*^Bu)], 34.0, 33.4 [2s, br, C^2^(*o*-^*t*^Bu)], 31.4 [s, C^2^*(p*-^*t*^Bu)].

### Preparation of [MoMnCp{μ-κ^2^_P,C_:η^3^-PMes*C(CO_2_Me)C(CO_2_Me)}(CO)_5_] (**2b.1**)

Dimethyl acetylenedicarboxylate (25 μL, 0.204 mmol) was added to a toluene solution (10 mL) of compound **1b** (0.040 g, 0.061 mmol), and the mixture was stirred at 333 K for 3 h to give an orange-brown solution. Workup was similar to the one described for **2a.1**. Elution with dichloromethane/petroleum ether (1/1) gave an orange fraction yielding compound **2b.1** as an orange microcrystalline solid (0.036 g, 77%). Anal. Calcd for C_34_H_40_MoMnO_9_P: C, 52.72; H, 5.21. Found: C, 52.43; H, 4.90. ^1^H NMR (400.13 MHz, CD_2_Cl_2_): *δ* 7.35, 7.26 (2s, br, 2 × 1H, C_6_H_2_), 5.56 (s, 5H, Cp), 3.64, 3.11 (2s, 2 × 3H, OMe), 1.60, 1.56 (2s, br, 2 × 9H, *o*-^*t*^Bu), 1.32 (s, 9H, *p*-^*t*^Bu). ^13^C{^1^H} NMR (100.63 MHz, CD_2_Cl_2_): *δ* 231.3 (d, *J*_CP_ = 8, MoCO), 231.0 (d, *J*_CP_ = 15, MoCO), 223.9 (s, br, 3MnCO), 174.4 (d, *J*_CP_ = 27, *C*O_2_Me), 164.4 (s, *C*O_2_Me), 162.0 [s, br, C^1^(C_6_H_2_)], 159.8 [s, br, C^4^(C_6_H_2_)], 152.2 [d, *J*_CP_ = 4, C^2,6^(C_6_H_2_)], 135.7 [s, C^6,2^(C_6_H_2_)], 123.5 [d, *J*_CP_ = 11, C^3,5^(C_6_H_2_)], 122.2 [d, *J*_CP_ = 7, C^5,3^(C_6_H_2_)], 118.6, 110.3 (2s, *C*CO_2_Me), 92.0 (s, Cp), 52.3, 51.4 (2s, OMe), 39.9, 39.7 [2s, br, C^1^(*o*-^*t*^Bu)], 35.1 [s, C^1^(*p*-^*t*^Bu)], 34.4, 33.3 [2s, br, C^2^(*o*-^*t*^Bu)], 31.3 [s, C^2^*(p*-^*t*^Bu)].

### Reaction of 1b with HC≡CCO_2_Me

Methyl propiolate (25 μL, 0.281 mmol) was added to a toluene solution (10 mL) of compound **1b** (0.060 g, 0.091 mmol), and the mixture was stirred at room temperature for 4 h to give an orange solution. Workup was similar to the one described for **2a.1**. Elution with dichloromethane/petroleum ether (1/20) gave a minor fraction of unreacted **1b**. Elution with dichloromethane/petroleum ether (1/3) gave orange and yellow fractions yielding isomers [MoMnCp{μ-κ^2^_P,C_:η^3^-PMes*CHC(CO_2_Me)}(CO)_5_] (**3b.1**) (0.040 g, 62%) and [MoMnCp{μ-κ^2^_P,C_:η^3^-PMes*C(CO_2_Me)CH}(CO)_5_] (**4b.1**) (0.020 g, 31%), as orange and yellow solids, respectively. The crystals of **4b.1** used in the X-ray diffraction study were grown by the slow diffusion of layers of diethyl ether and petroleum ether into a concentrated dichloromethane solution of the complex at 253 K. *Data for compound**3b.1***: Anal. Calcd for C_32_H_38_MoMnO_7_P: C, 53.64; H, 5.35. Found: C, 53.78; H, 5.18. ^1^H NMR (300.13 MHz, CD_2_Cl_2_): *δ* 7.37, 7.26 (2s, br, 2 × 1H, C_6_H_2_), 5.66 (d, *J*_HP_ = 5, 1H, PCH), 5.47 (s, 5H, Cp), 3.62 (s, 3H, OMe), 1.57, 1.54 (2s, br, 2 × 9H, *o*-^*t*^Bu), 1.27 (s, 9H, *p*-^*t*^Bu). ^13^C{^1^H} NMR (100.63 MHz, CD_2_Cl_2_): *δ* 234.2 (d, *J*_CP_ = 14, MoCO), 232.4 (d, *J*_CP_ = 9, MoCO), 225.0 (s, br, 3MnCO), 175.5 (d, *J*_CP_ = 29, *C*O_2_Me), 159.8 [s, br, C^1^(C_6_H_2_)], 158.5 [s, br, C^4^(C_6_H_2_)], 152.1 [d, *J*_CP_ = 4, C^2,6^(C_6_H_2_)], 125.0 [s, br, C^6,2^(C_6_H_2_)], 122.6 [d, *J*_CP_ = 2, C^3,5^(C_6_H_2_)], 122.1 (s, br, PCH), 121.8 [d, *J*_CP_ = 6, C^5,3^(C_6_H_2_)], 117.5 (d, *J*_CP_ = 12, *C*CO_2_Me), 90.6 (s, Cp), 52.1 (s, OMe), 39.9, 39.3 [2s, br, C^1^(*o*-^*t*^Bu)], 35.2 [s, br, C^2^(*o*-*t*Bu)], 35.0 [s, C^1^*(p*-^*t*^Bu)], 32.9 [s, br, C^2^(*o*-^*t*^Bu)], 31.1 [s, C^2^*(p*-^*t*^Bu)]. *Data for compound**4b.1***: Anal. Calcd for C_32_H_38_MoMnO_7_P: C, 53.64; H, 5.35. Found: C, 53.42; H, 4.64. ^1^H NMR (300.13 MHz, CD_2_Cl_2_): *δ* 7.39 (d, *J*_HP_ = 47, 1H, PCCH), 7.35 (dd, *J*_HP_ = 5, *J*_HH_ = 2, 1H, C_6_H_2_), 7.25 (t, *J*_HP_ = *J*_HH_ = 2, 1H, C_6_H_2_), 5.52 (s, 5H, Cp), 3.10 (s, 3H, OMe), 1.64, 1.56, 1.27 (3s, 3 × 9H, ^*t*^Bu). ^13^C{^1^H} NMR (100.63 MHz, CD_2_Cl_2_): *δ* 233.4 (d, *J*_CP_ = 7, MoCO), 230.9 (d, *J*_CP_ = 15, MoCO), 224.7 (s, br, 3MnCO), 165.5 (s, *C*O_2_Me), 161.4 [d, *J*_CP_ = 6, C^2,6^(C_6_H_2_)], 159.2 [s, br, C^4^(C_6_H_2_)], 151.9 [d, *J*_CP_ = 4, C^6,2^(C_6_H_2_)], 130.6 [d, *J*_CP_ = 12, C^1^(C_6_H_2_)], 123.5 [d, *J*_CP_ = 11, C^3,5^(C_6_H_2_)], 122.2 [d, *J*_CP_ = 9, C^5,3^(C_6_H_2_)], 120.9 (d, *J*_CP_ = 4, *C*CO_2_Me), 110.6 (s, br, PC*C*H), 90.5 (s, Cp), 51.0 (s, OMe), 39.9, 39.5, 35.1 [3s, C^1^(^*t*^Bu)], 34.1 [s, C^2^(^*t*^Bu)], 33.4 [d, *J*_CP_ = 4, C^2^(^*t*^Bu)], 31.3 [s, C^2^(^*t*^Bu)].

### Preparation of [MoReCp(μ-κ^2^_P,C_:η^3^-PMes*CHC^*t*^Bu}(CO)_5_] (**3a.3**)

*Tert*-butylacetylene (52 μL, 0.422 mmol) was added to a toluene solution (8 mL) of compound **1a** (0.020 g, 0.025 mmol), and the mixture was stirred at room temperature for 72 h to give an orange solution. Workup was similar to the one described for **2a.1**. Elution with dichloromethane/petroleum ether (1/8) gave a minor fraction of unreacted **1a**. Elution with dichloromethane/petroleum ether (1/3) gave an orange fraction yielding compound **3a.3** as an orange microcrystalline solid (0.006 g, 29%). Anal. Calcd for C_34_H_44_MoO_5_PRe: C, 48.28; H, 5.24. Found: C, 48.18; H, 4.73. ^1^H NMR (400.13 MHz, CD_2_Cl_2_): *δ* 7.35 (t, *J*_HP_ = *J*_HH_ = 3, 1H, C_6_H_2_), 7.24 (dd, *J*_HP_ = 4, *J*_HH_ = 2, 1H, C_6_H_2_), 6.96 (d, *J*_HP_ = 7, 1H, PCH), 5.02 (s, 5H, Cp), 1.63, 1.49, 1.30, 1.19 (4s, 4 × 9H, ^*t*^Bu). ^13^C{^1^H} NMR (100.63 MHz, CD_2_Cl_2_): *δ* 229.6 (d, *J*_CP_ = 8, MoCO), 225.9 (s, MoCO), 200.0 (d, *J*_CP_ = 17, 3ReCO), 162.5 [d, *J*_CP_ = 14, C^1^(C_6_H_2_)], 159.7 [d, *J*_CP_ = 6, C^2,6^(C_6_H_2_)], 159.6 [s, C^4^(C_6_H_2_)], 151.6 [d, *J*_CP_ = 3, C^6,2^(C_6_H_2_)], 124.8 [d, *J*_CP_ = 7, C^3,5^(C_6_H_2_)], 122.0 [d, *J*_CP_ = 9, C^5,3^(C_6_H_2_)], 117.7 (s, br, *C^t^*Bu), 103.7 (s, br, PCH), 90.8 (s, Cp), 43.1 [d, *J*_CP_ = 22, C^1^(^*t*^Bu)], 40.5, 39.8 [2s, C^1^(^*t*^Bu)], 35.2 [s, C^2^(^*t*^Bu)], 35.0 [s, C^1^(^*t*^Bu)], 33.23 [s, C^2^(^*t*^Bu)], 33.18 [d, *J*_CP_ = 4, C^2^(^*t*^Bu)], 31.1 [s, C^2^(^*t*^Bu)].

### Preparation of [MoReCp{μ-η^3^:κ^1^_C_-PMes*CHC(CO_2_Me)}(CO)_5_{CN(*p*-C_6_H_4_OMe)}_2_] (**5**)

Neat CN(*p*-C_6_H_4_OMe) (0.008 g, 0.060 mmol) was added to a toluene solution (8 mL) of a ca. 5:1 mixture of isomers **3a.1** and **4a.1**,^6^ (0.020 g, 0.024 mmol), and the mixture was stirred at 273 K for 3 h to give a yellow solution. Workup was similar to the one described for **2a.1**. Elution with dichloromethane/petroleum ether (1/3) gave a yellow fraction yielding compound **5** as a yellow solid (0.018 g, 69%). The crystals of **5** used in the X-ray diffraction study were grown by the slow diffusion of layers of diethyl ether and petroleum ether into a concentrated dichloromethane solution of the complex at 253 K. Anal. Calcd for C_48_H_52_MoN_2_O_9_PRe: C, 51.75; H, 4.70; N, 2.51. Found: C, 51.41; H, 4.45; N, 2.56. ^1^H NMR (300.13 MHz, CD_2_Cl_2_): *δ* 7.50–7.47 (m, 4H, C_6_H_4_), 7.28 (d, *J*_HH_ = 2, 1H, C_6_H_2_), 7.12 (s, br, 1H, C_6_H_2_), 6.97–6.92 (m, 4H, C_6_H_4_), 5.99 (d, *J*_PH_ = 4, 1H, PCH), 4.52 (s, 5H, Cp), 3.84, 3.83, 3.43 (3s, 3 × 3H, OMe), 1.72, 1.65, 1.26 (3 s, 3 × 9H, ^*t*^Bu). ^13^C{^1^H} NMR (100.63 MHz, CD_2_Cl_2_): *δ* 243.6 (s, MoCO), 241.3 (d, *J*_CP_ = 19, MoCO), 197.3 (s, br, ReCO), 190.0 (d, *J*_CP_ = 32, ReCO), 185.0 (s, br, ReCO), 182.0 (d, *J*_CP_ = 20, *C*O_2_Me), 160.5 [s, C^4^(C_6_H_4_)], 158.7, 158.6 [2s, C^2,6^(C_6_H_2_)], 156.5 [s, C^4^(C_6_H_2_)], 152.2 (s, br, *C*CO_2_Me), 146.9 [s, C^1^(C_6_H_4_)], 146.1, 145.4 (2s, br, CN), 141.7 [d, *J*_CP_ = 76, C^1^(C_6_H_2_)], 128.6 [d, *J*_CP_ = 5, C^2^(C_6_H_4_)], 123.7, 121.0 [2s, C^3,5^(C_6_H_2_)], 115.0 [s, C^3^(C_6_H_4_)], 95.5 (d, *J*_CP_ = 54, PCH), 93.1 (s, Cp), 56.1 (s, 2OMe), 51.0 (s, OMe), 40.2, 39.8 [2s, C^1^(^*t*^Bu)], 36.4 [d, *J*_CP_ = 4, C^2^(^*t*^Bu)], 34.7 [s, C^1^(^*t*^Bu)], 34.0 [d, *J*_CP_ = 11, C^2^(^*t*^Bu)], 31.1 [s, C^2^(^*t*^Bu)].

### Reaction of **1a** with SCNPh

Phenyl isothiocyanate (9 μL, 0.075 mmol) was added to a toluene solution (8 mL) of compound **1a** (0.050 g, 0.063 mmol), and the solution was refluxed for 3 h to give a brown solution. Workup was similar to the one described for **2a.1**. Elution with dichloromethane/petroleum ether (1/6) gave a minor fraction of [MoReCp(μ-H){μ-P(CH_2_CMe_2_)C_6_H_2_^*t*^Bu_2_}(CO)_6_],^6^ then minor gray and yellow fractions containing uncharacterized species, followed by major orange and yellow fractions. The solvent was removed from the orange fraction, and the residue was recrystallized by the slow diffusion of a layer of petroleum ether into a concentrated diethyl ether solution of that residue, to give compound [MoReCp(μ-η^2^:κ^1^_S_-SPMes*)(CO)_5_(CNPh)] (**7**) as orange crystals suitable for the X-ray study (0.018 g, 32%). Removal of the solvent from the yellow fraction yielded pure complex [MoReCp{μ-κ^2^_P,S_:κ^2^_P,S_-PMes*C(NPh)S}(CO)_5_] (**6**) as a yellow microcrystalline solid (0.012 g, 21%). The crystals of **6** used in the X-ray diffraction study were grown by the slow diffusion of a layer of petroleum ether into a concentrated toluene solution of the complex at 253 K. *Data for compound**6***: Anal. Calcd for C_35_H_39_MoNO_5_PReS: C, 46.77; H, 4.37; N, 1.56; S, 3.57. Found: C, 46.50; H, 4.06; N, 1.33; S, 3.31. ^1^H NMR (400.13 MHz, CD_2_Cl_2_): *δ* 7.36–7.33 [m, 4H, C_6_H_2_ and H^3^(Ph)], 7.09 [t, *J*_HH_ = 7, 1H, H^4^(Ph)], 6.87 [d, *J*_HH_ = 8, 2H, H^2^(Ph)], 5.19 (s, 5H, Cp), 1.44 (s, 18H, *o*-^*t*^Bu), 1.34 (s, 9H, *p*-^*t*^Bu). ^13^C{^1^H} NMR (100.63 MHz, CD_2_Cl_2_): *δ* 232.6 (d, *J*_CP_ = 11, MoCO), 229.3 (s, MoCO), 197.8 (s, br, 3ReCO), 177.3 (d, *J*_CP_ = 27, PCN), 151.9 [s, C^4^(C_6_H_2_)], 145.3 [d, *J*_CP_ = 16, C^2^(C_6_H_2_)], 129.8 [s, C^2^(Ph)], 125.2 [s, C^4^(Ph)], 123.0 [s, C^3^(Ph)], 122.1 [d, *J*_CP_ = 8, C^3^(C_6_H_2_)], 115.4 [d, *J*_CP_ = 10, C^1^(C_6_H_2_)], 94.2 (s, Cp), 35.0 [s, C^1^(*o*-^*t*^Bu)], 34.0 [s, C^2^(*o*-^*t*^Bu)], 32.0 [s, C^1^(*p*-^*t*^Bu)], 31.1 [s, C^2^(*p*-^*t*^Bu)]; the resonance for atom C^1^(Ph) could not be identified in the spectrum. *Data for compound**7***: Anal. Calcd for C_35_H_39_MoNO_5_PReS: C, 46.77; H, 4.37; N, 1.56; S, 3.57. Found: C, 46.41; H, 3.96; N, 1.35; S, 3.23. ^1^H NMR (400.13 MHz, CD_2_Cl_2_): *δ* 7.41 (s, br, 7H, C_6_H_2_ and Ph), 4.88 (s, 5H, Cp), 1.72, 1.57 (2s, br, 2 × 9H, *o*-^*t*^Bu), 1.25 (s, 9H, *p*-^*t*^Bu). ^13^C{^1^H} NMR (100.63 MHz, CD_2_Cl_2_): *δ* 242.2 (d, *J*_CP_ = 15, MoCO), 232.1 (s, MoCO), 190.9 (s, ReCO), 188.7 (d, *J*_CP_ = 3, ReCO), 187.9 (s, ReCO), 156.9 [s, br, C^2^(C_6_H_2_)], 148.1 [d, *J*_CP_ = 97, C^1^(C_6_H_2_)], 148.1 [s, C^4^(C_6_H_2_)], 130.0 [s, C^4^(Ph)], 129.7 [s, C^2^(Ph)], 126.9 [s, C^3^(Ph)], 123.6 [s, br, C^3^(C_6_H_2_)], 93.6 (s, Cp), 40.6, 39.6 [2s, br, C^1^(*o*-^*t*^Bu)], 36.0 [s, br, C^2^(*o*-^*t*^Bu)], 34.7 [s, C^1^(*p*-^*t*^Bu)], 33.6 [s, br, C^2^(*o*-^*t*^Bu)], 31.1 [s, C^2^(*p*-^*t*^Bu)]; the resonances of the N-bound carbons could not be identified in the spectrum.

### X-ray Structure Determination of Compounds **4b.1**, **5**, **6**, and **7**

Data collection for these compounds was performed at ca. 150 K on an Oxford Diffraction Xcalibur Nova single crystal diffractometer, using Cu Kα radiation. Images were collected at a 62 mm fixed crystal-detector distance using the oscillation method, with 1.0–1.3° oscillation and variable exposure time per image. The data collection strategy was calculated with the program *CrysAlis Pro CCD*,^34^ and data reduction and cell refinements were performed with the program *CrysAlis Pro RED*.^34^ An empirical absorption correction was applied using the SCALE3 ABSPACK algorithm as implemented in the program *CrysAlis Pro RED*. Using the program suite WinGX,^35^ the structures were solved by Patterson interpretation and phase expansion using SHELXL2018/3,^36^ and refined with full-matrix least squares on *F*^2^ using SHELXL2018/3. In general, all nonhydrogen atoms were refined anisotropically except for atoms involved in disorder, which were refined isotropically to prevent their temperature factors from becoming nonpositive definite, and all hydrogen atoms were geometrically placed and refined using a riding model, to give the residuals shown in Table S1. For compound **4b.1**, the Cp ligand was disordered, and satisfactorily modeled over two sites with 0.5/0.5 occupancies; moreover, one of the ^*t*^Bu groups displayed incipient disorder, but this could not be modeled.

### Computational Details

DFT calculations on compound **7** and some isomers and related species were carried out using the GAUSSIAN16 package,^37^ and the M06L functional.^38^ A pruned numerical integration grid (99,590) was used for all the calculations *via* the keyword Int = Ultrafine. Effective core potentials and their associated double-ζ LANL2DZ basis set were used for Mo and Re atoms.^39^ The light elements (P, S, O, C, N, and H) were described with the 6-31G* basis.^40^ Geometry optimizations were performed under no symmetry restrictions, using initial coordinates derived from the corresponding or related X-ray data. Frequency analyses were performed for all the stationary points to ensure that a minimum structure with no imaginary frequencies was achieved (one imaginary frequency for transition states).

## Supporting Information Available

Crystal data for compounds **4b.1**, and **5** to **7** (CCDC 2258005 to 2258008), IR and NMR spectra for all new compounds, and results of DFT calculations on compound **7** and related species (PDF)
Cartesian coordinates and relative Gibbs free energies of all DFT-computed species (XYZ)

## Supplementary Material

om3c00242_si_001.pdf

om3c00242_si_002.xyz
