# Supplementary material for: Cycloaddition and C–S Bond Cleavage Processes in Reactions of Heterometallic Phosphinidene-Bridged MoRe and MoMn Complexes with Alkynes and Phenyl Isothiocyanate
Source: Organometallics. 2023 Jul 7;42(15):2052–64. doi: 10.1021/acs.organomet.3c00242 (PMC10431387; doi:10.1021/acs.organomet.3c00242)
Supplement: Supplementary file 1 — om3c00242_si_001.pdf [file om3c00242_si_001.pdf]

Supporting Information

Cycloaddition and C–S Bond Cleavage Processes in Reactions of Heterometallic Phosphinidene-Bridged MoRe and MoMn Complexes with Alkynes and Phenyl Isothiocyanate.

M. Angeles Alvarez, M. Esther García, Daniel García-Vivó,* Miguel A. Ruiz,* and Patricia Vega

Departamento de Química Orgánica e Inorgánica/IUQOEM, Universidad de Oviedo, E-33071 Oviedo, Spain.

Corresponding Author E-mail: garciavdaniel@uniovi.es (D.G.V.), mara@uniovi.es (M.A.R).

Table of Contents

		page
1.	Crystal data for new compounds (Table S1)	S-3
2.	IR and NMR data for compound **2a.1** (Figs. S1 to S4)	S-4
3.	IR and NMR data for compound **2a.2** (Figs. S5 to S8)	S-6
4.	IR and NMR data for compound **2b.1** (Figs. S9 to S12)	S-8
5.	IR and NMR data for compound **3a.3** (Figs. S13 to S16)	S-10
6.	IR and NMR data for compound **3b.1** (Figs. S17 to S20)	S-12
7.	IR and NMR data for compound **4b.1** (Figs. S21 to S24)	S-14
8.	IR and NMR data for compound **5** (Figs. S25 to S28)	S-16
9.	IR and NMR data for compound **6** (Figs. S29 to S32)	S-18
10.	IR and NMR data for compound **7** (Figs. S33 to S36)	S-20
11.	DFT optimized structures of compound **7** and related isomers (Figure S37).	S-22

Table S1. Crystal Data for New Compounds

	**4b.1·**	**5**	**6**	**7**
mol formula	$C_{32}H_{38}MnMoO_7P$	$C_{48}H_{52}MoN_2O_9PRe$	$C_{35}H_{39}MoNO_5PReS$	$C_{35}H_{39}MoNO_5PReS$
mol wt	716.47	1114.04	898.85	898.85
cryst syst	triclinic	monoclinic	triclinic	monoclinic
space group	*P*–1	*P*2$_1$/c	*P*–1	*P*2$_1$/n
radiation (λ, Å)	1.54184	1.54184	1.54184	1.54184
a, Å	12.0708(4)	17.5295(10)	9.4930(3)	14.8048(2)
b, Å	12.1611(4)	9.9189(5)	11.6066(3)	14.6065(3)
c, Å	13.2138(5)	29.058(2)	16.5142(6)	16.4392(3)
α, deg	63.946(4)	90	96.232(3)	90
β, deg	68.040(3)	106.643(7)	103.115(3)	91.979(1)
γ, deg	84.127(3)	90	101.659(3)	90
V, Å^3	1611.7(1)	4840.8(5)	1712.6(1)	3552.8(1)
Z	2	4	2	4
calcd density, g cm^{-3}	1.476	1.529	1.743	1.680
absorp coeff, mm^{-1}	7.211	7.694	11.154	10.754
temperature, K	155(3)	151(2)	130.0(1)	150.1(1)
θ range (deg)	3.96 / 69.60	4.67 / 70.20	4.44 / 69.50	3.95 / 69.51
index ranges (*h*, *k*, *l*)	–14, 14; –14, 10<br>–16, 15	–21, 21; –11, 11<br>–35, 30	–11, 11; –13, 14<br>–19, 20	–15, 17; –17, 17<br>–18, 19
no. of reflns collected	15546	18618	16302	20533
no. of indep reflns (R_{int})	5972(0.0284)	8844(0.0897)	6337(0.0871)	6618(0.0638)
reflns with $I > 2\sigma(I)$	5586	6218	6154	5855
R indexes [data with $I > 2\sigma(I)$][a]	R_1 = 0.0343<br>wR_2 = 0.0747[b]	R_1 = 0.0843<br>wR_2 = 0.2130[c]	R_1 = 0.0503<br>wR_2 = 0.1350[d]	R_1 = 0.0436<br>wR_2 = 0.1188[e]
R indexes (all data)[a]	R_1 = 0.0887<br>wR_2 = 0.0904[b]	R_1 = 0.1092<br>wR_2 = 0.2398[c]	R_1 = 0.0511<br>wR_2 = 0.1362[d]	R_1 = 0.0493<br>wR_2 = 0.1263[e]
GOF	1.071	1.051	1.079	1.073
no. of restraints/params	0 / 366	1 / 683	0 / 415	0 / 415
$\Delta\rho$(max., min.), eÅ^{-3}	1.219 / –0.7828	2.685 / – 1.375	2.082 / –3.956	1.773 / –1.886
CCDC deposition no	2258005	2258006	2258007	2258008

[a] $R = \Sigma||F_o| - |F_c|| / \Sigma|F_o|$. $wR = [\Sigma w(|F_o|^2 - |F_c|^2)^2 / \Sigma w|F_o|^2]^{1/2}$. $w = 1/[\sigma^2(F_o^2) + (aP)^2 + bP]$ where $P = (F_o^2 + 2F_c^2)/3$. [b] *a* = 0.0402, *b* = 2.2372. [c] *a* = 0.1254, *b* = 2.7884. [d] *a* = 0.0974, *b* = 0.8156. [e] *a* = 0.0794, *b* = 0.2034.

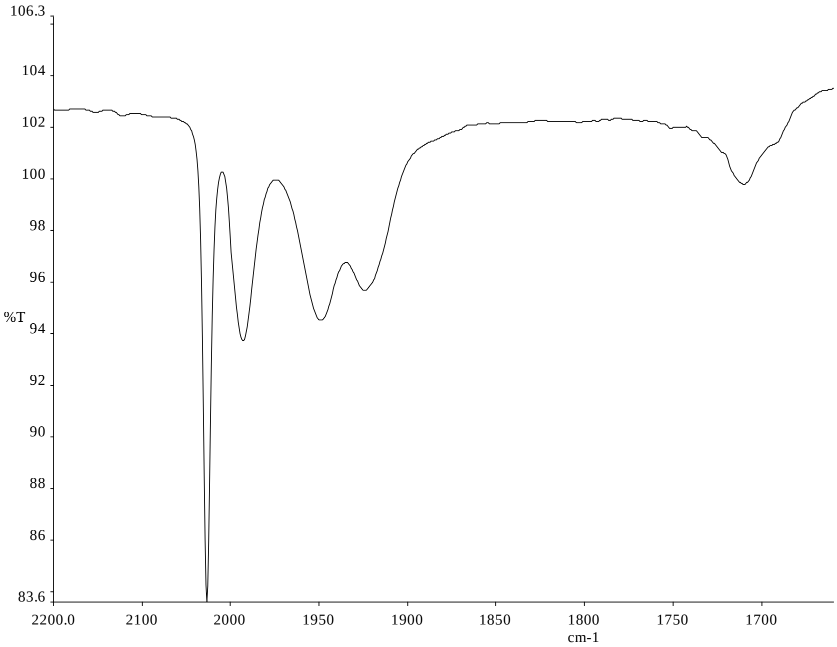

Figure S1. IR spectrum of compound **2a.1** in dichloromethane solution.

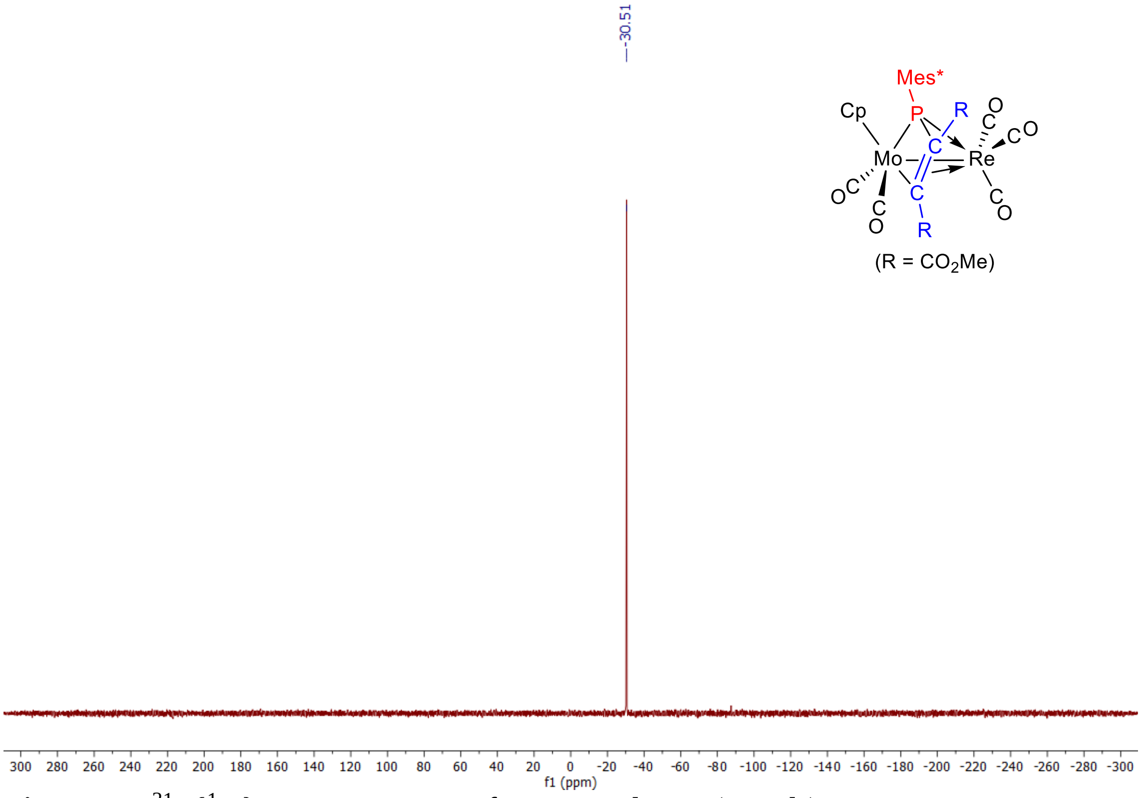

Figure S2. $^{31}P\{^{1}H\}$ NMR spectrum of compound **2a.1** ($CDCl_2$).

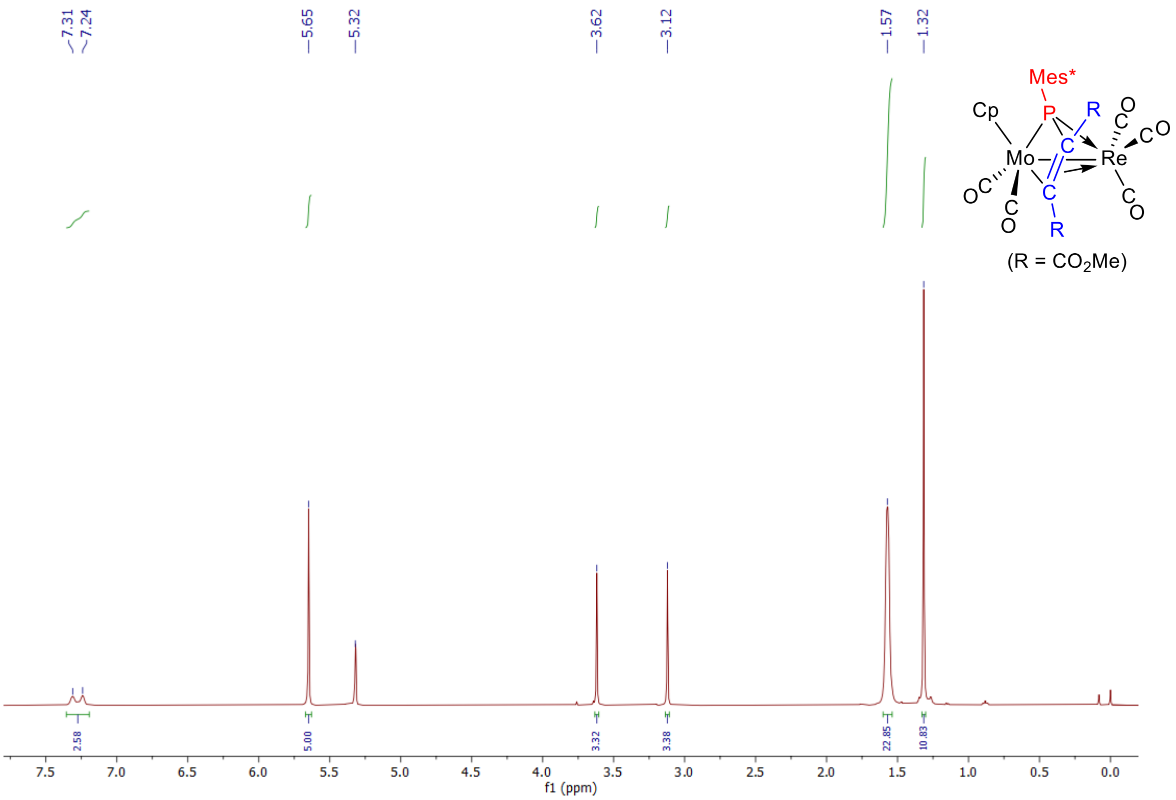

Figure S3. ^{1}H NMR spectrum of compound **2a.1** (CD_2Cl_2).

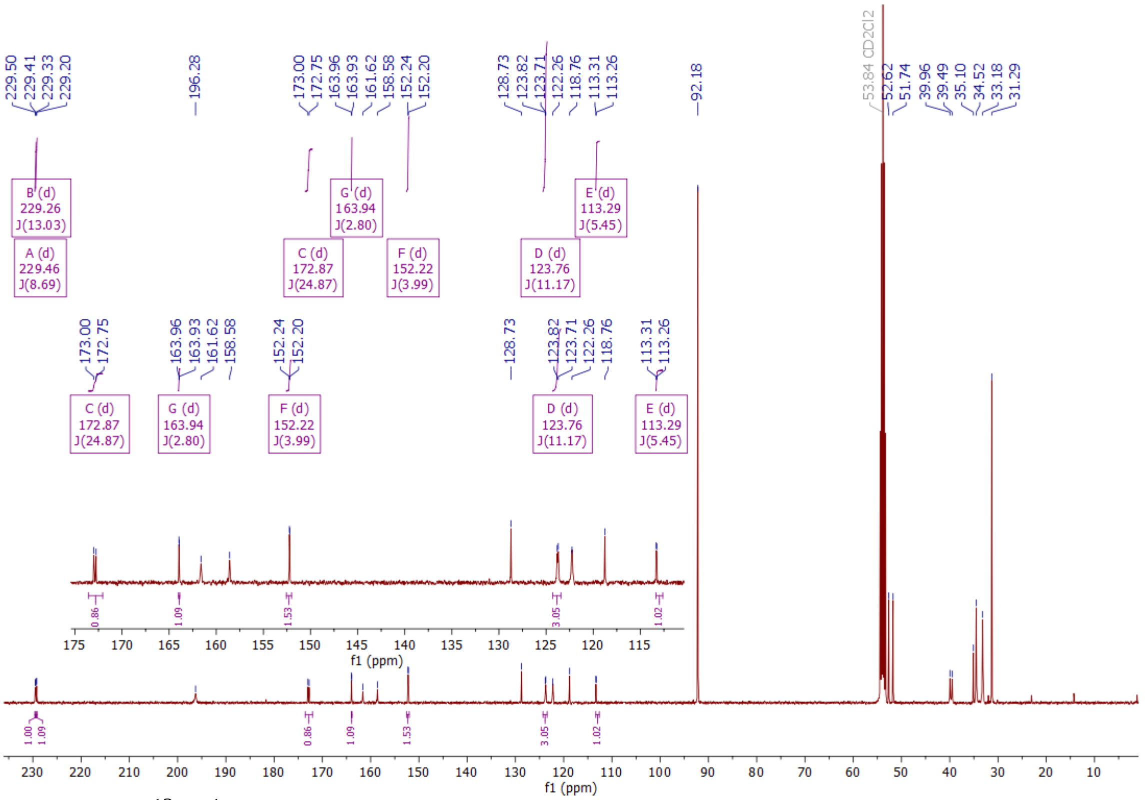

Figure S4. $^{13}C\{^{1}H\}$ NMR spectrum of compound **2a.1** (CD_2Cl_2).

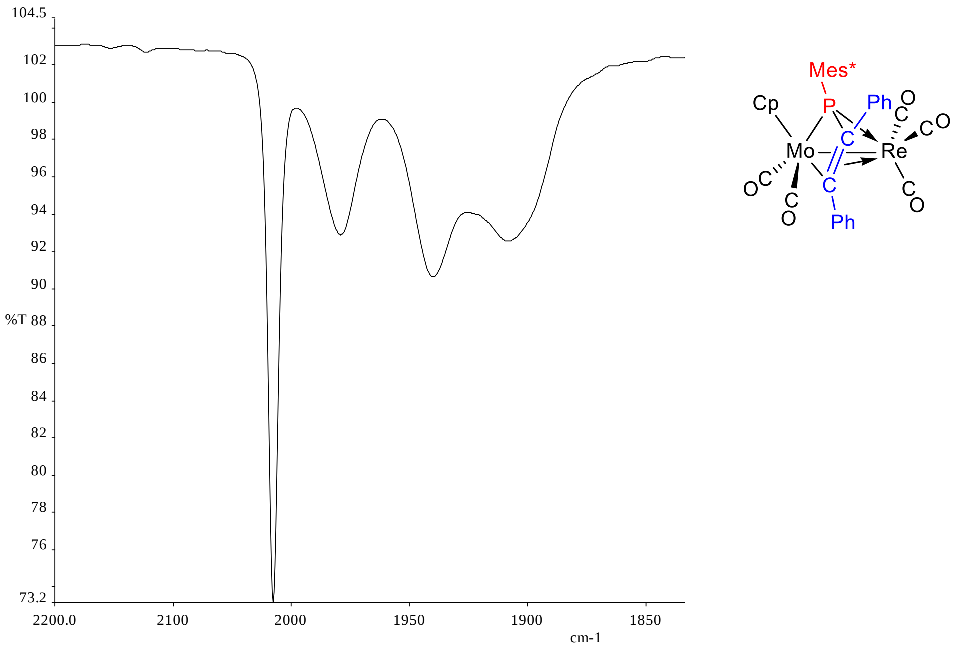

Figure S5. IR spectrum of compound **2a.2** in dichloromethane solution.

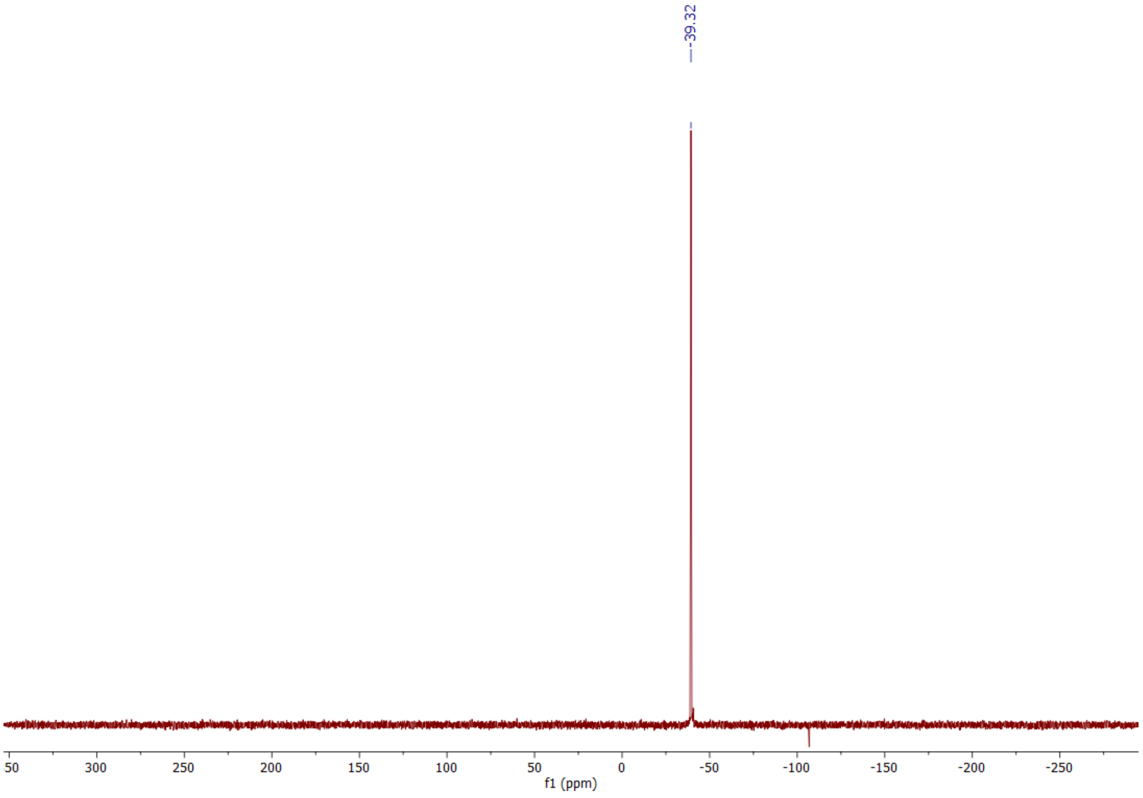

Figure S6. $^{31}P\{^{1}H\}$ NMR spectrum of compound **2a.2** (CDCl₂).

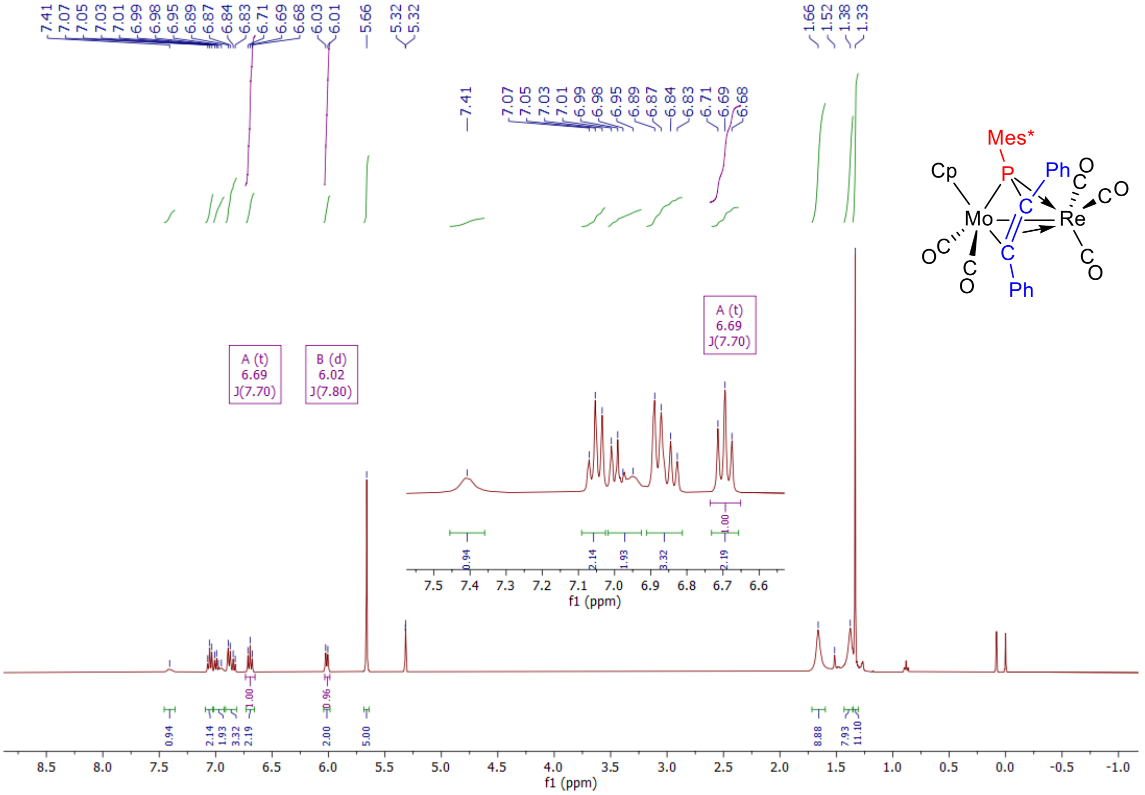

Figure S7. ^{1}H NMR spectrum of compound **2a.2** (CD_2Cl_2).

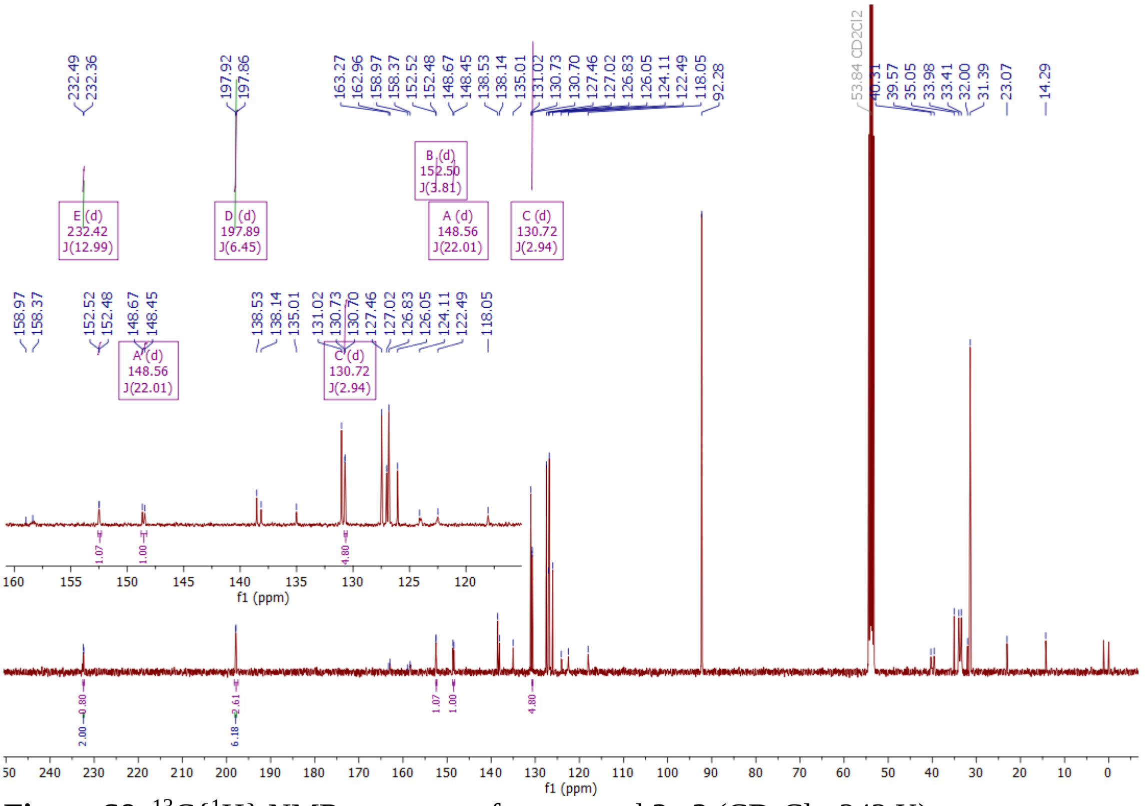

Figure S8. $^{13}C\{^{1}H\}$ NMR spectrum of compound **2a.2** (CD_2Cl_2, 243 K).

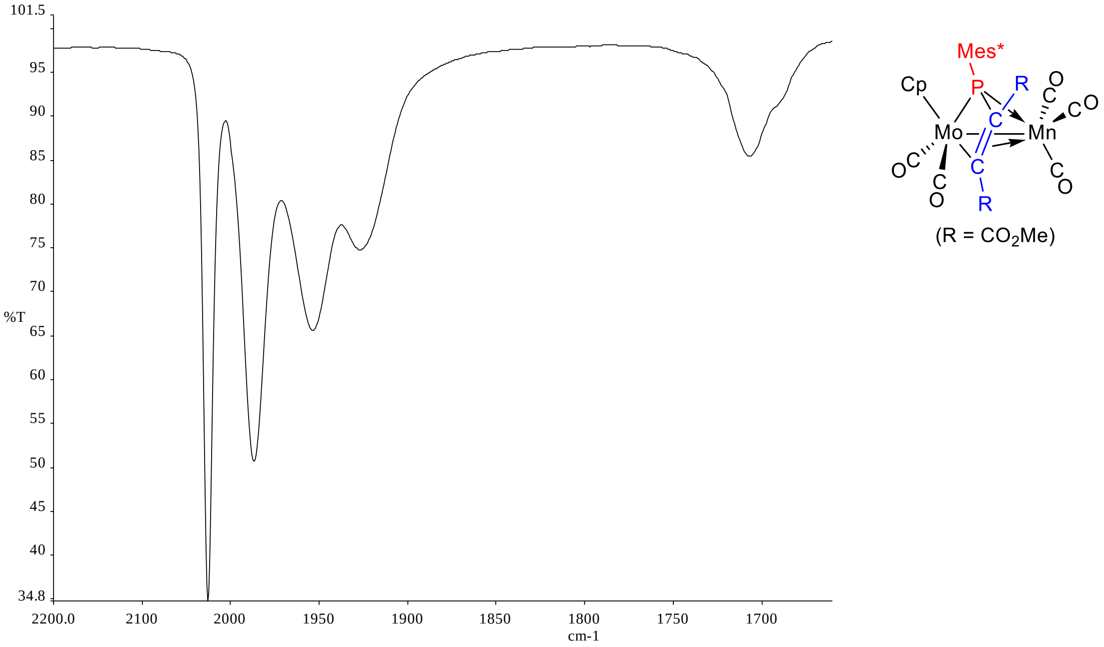

Figure S9. IR spectrum of compound **2b.1** in dichloromethane solution.

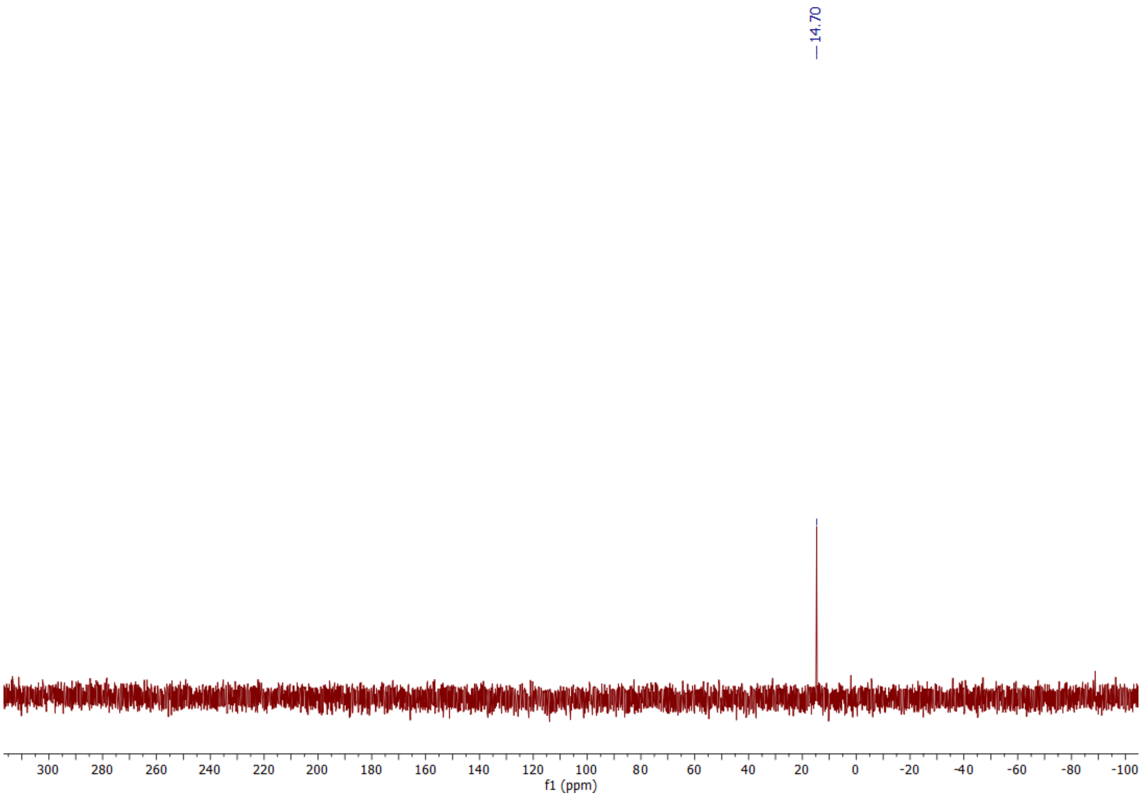

Figure S10. $^{31}P\{^{1}H\}$ NMR spectrum of compound **2b.1** (CD_2Cl_2).

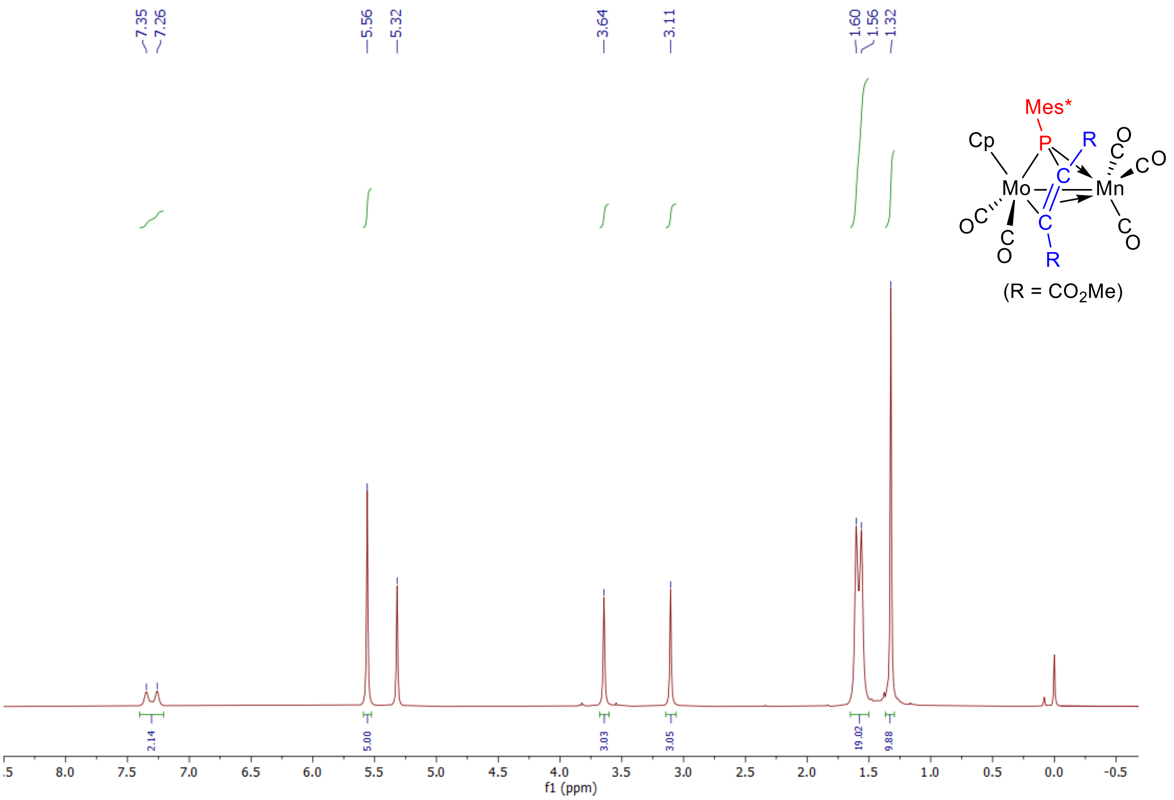

(R = CO_2Me)

Figure S11. ^{1}H NMR spectrum of compound **2b.1** (CD_2Cl_2).

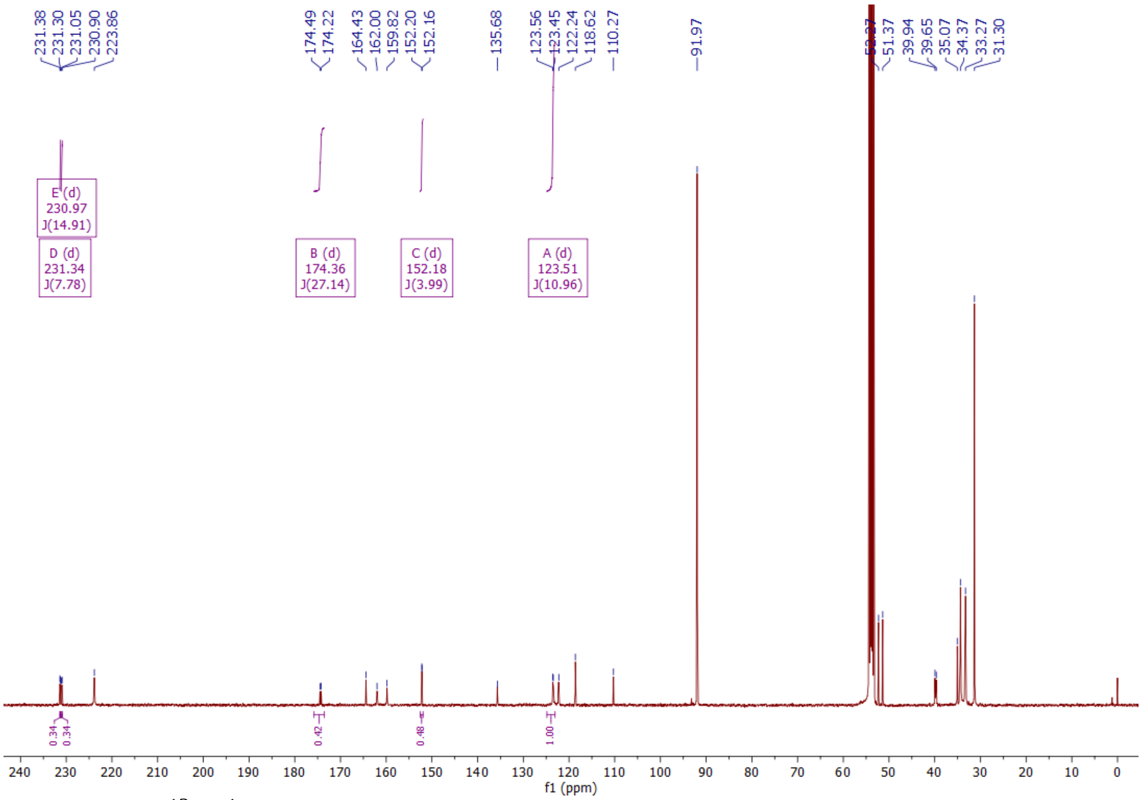

Figure S12. $^{13}C\{^{1}H\}$ NMR spectrum of compound **2b.1** (CD_2Cl_2).

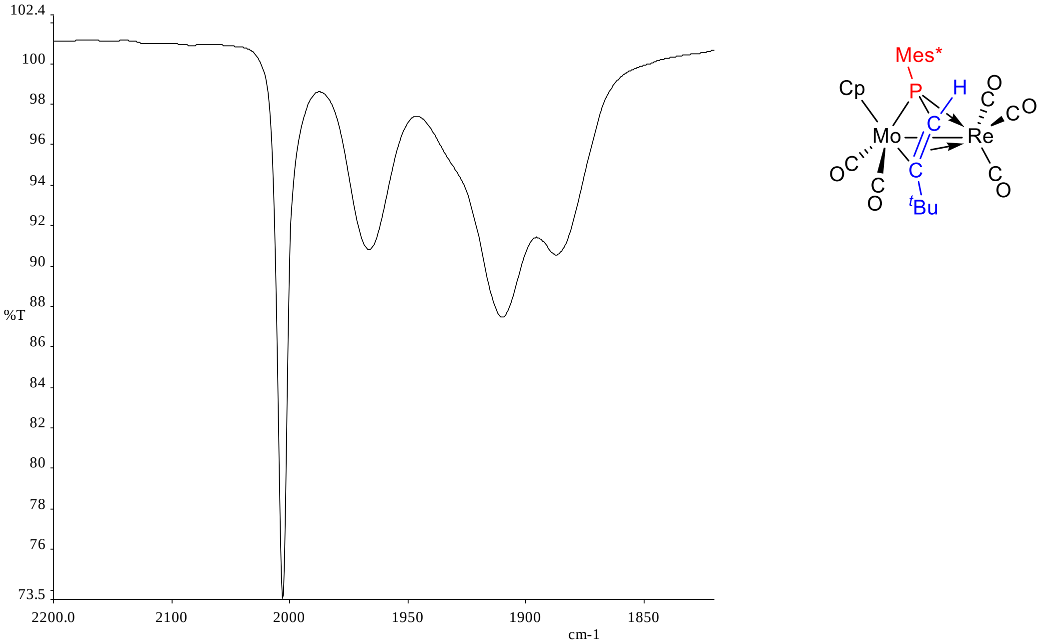

Figure S13. IR spectrum of compound **3a.3** in dichloromethane solution.

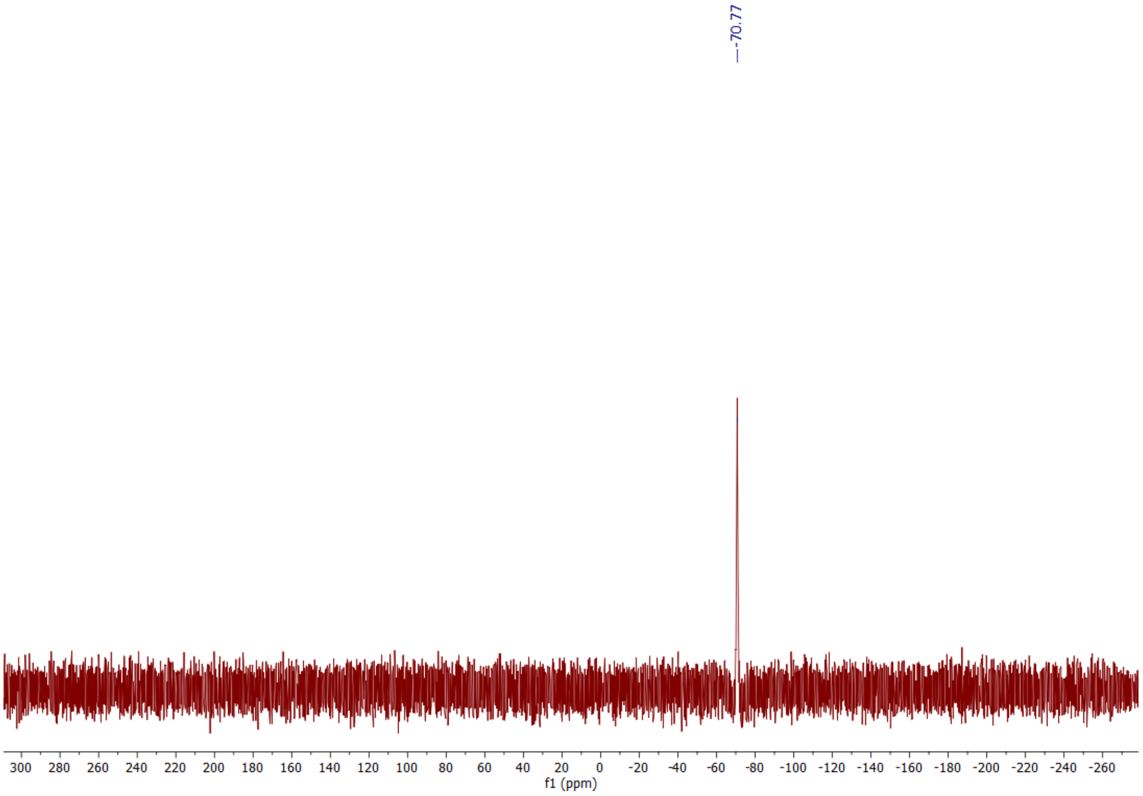

Figure S14. $^{31}P\{^{1}H\}$ NMR spectrum of compound **3a.3** (CD_2Cl_2).

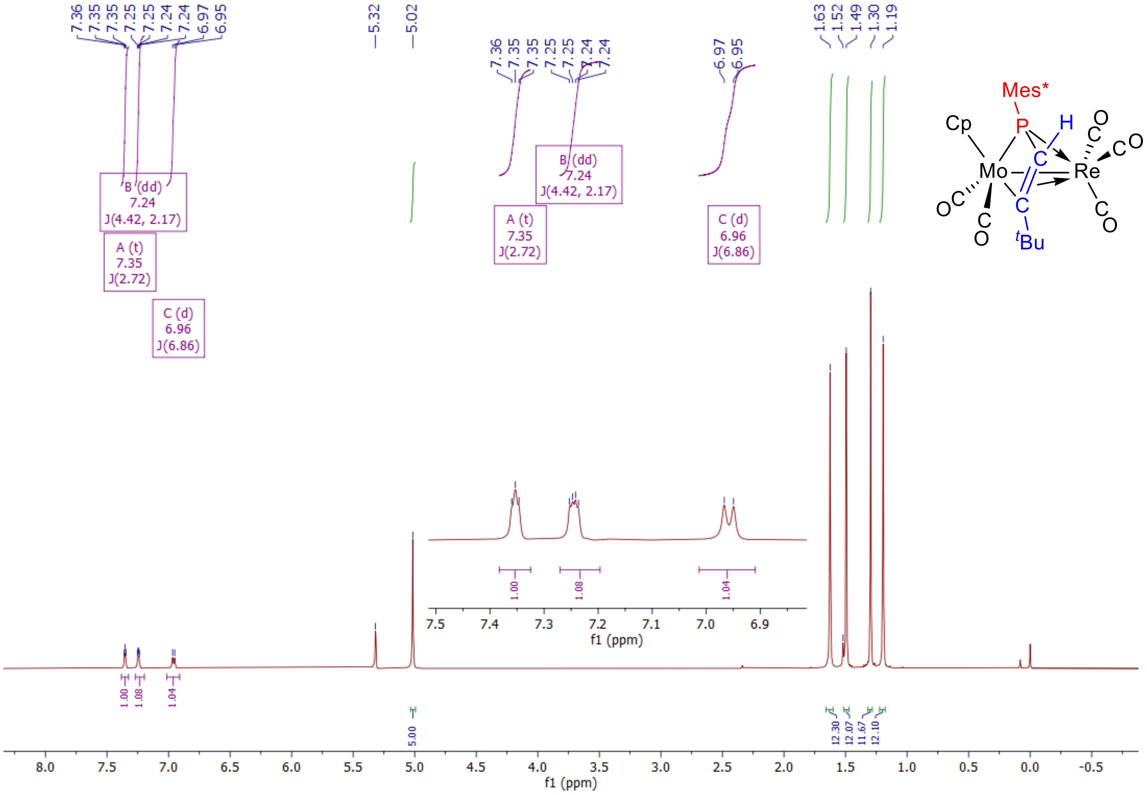

Figure S15. ^{1}H NMR spectrum of compound **3a.3** (CD_2Cl_2).

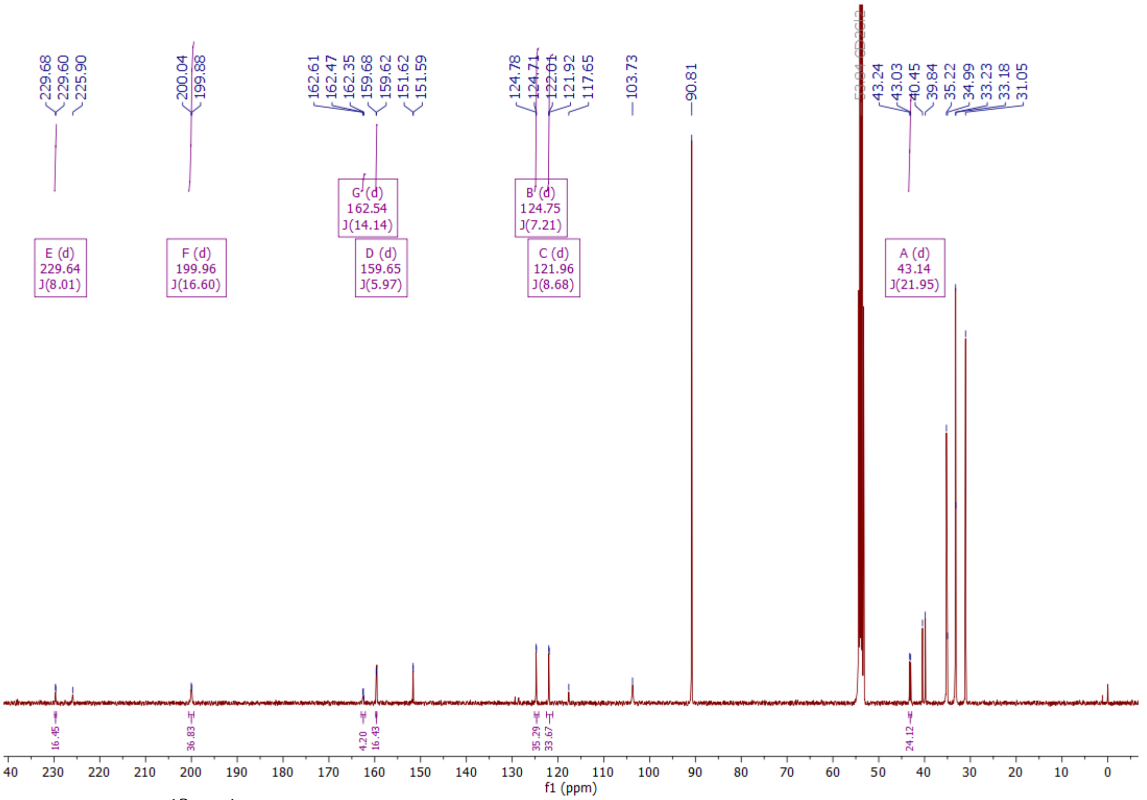

Figure S16. $^{13}C\{^{1}H\}$ NMR spectrum of compound **3a.3** (CD_2Cl_2).

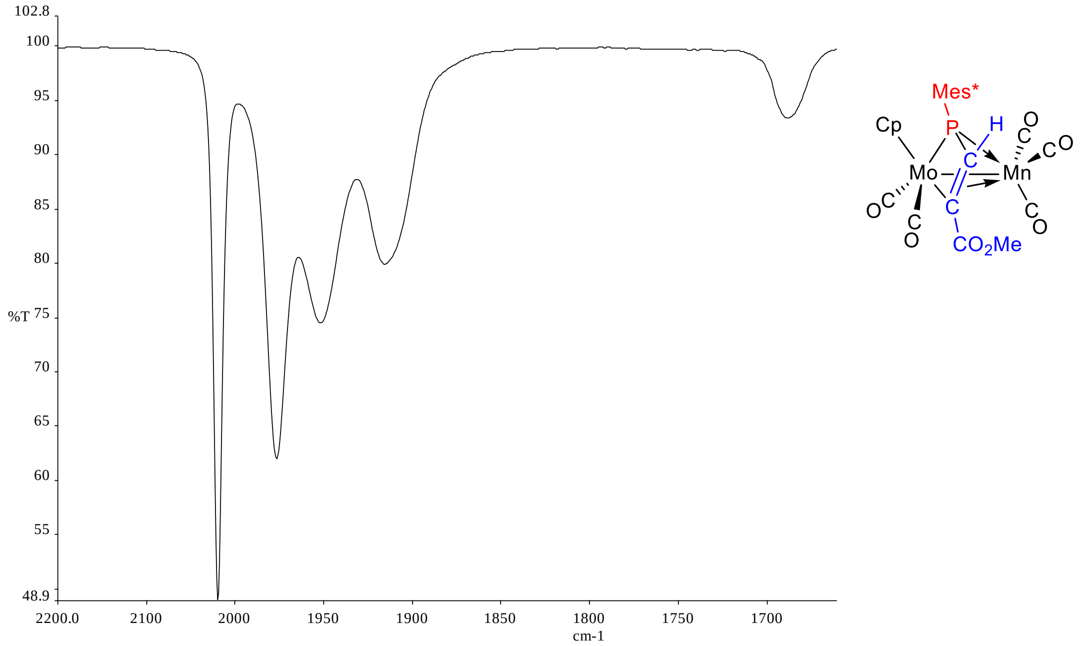

Figure S17. IR spectrum of compound **3b.1** in dichloromethane solution.

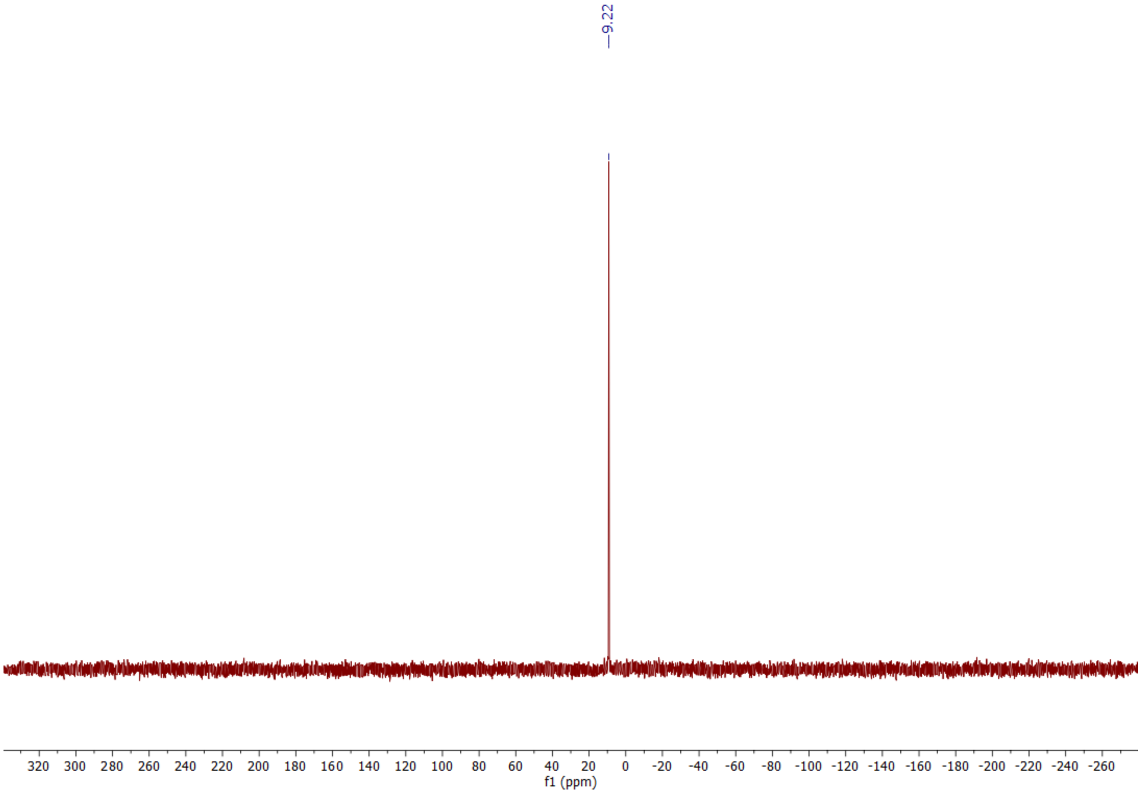

Figure S18. $^{31}P\{^1H\}$ NMR spectrum of compound **3b.1** (CD_2Cl_2).

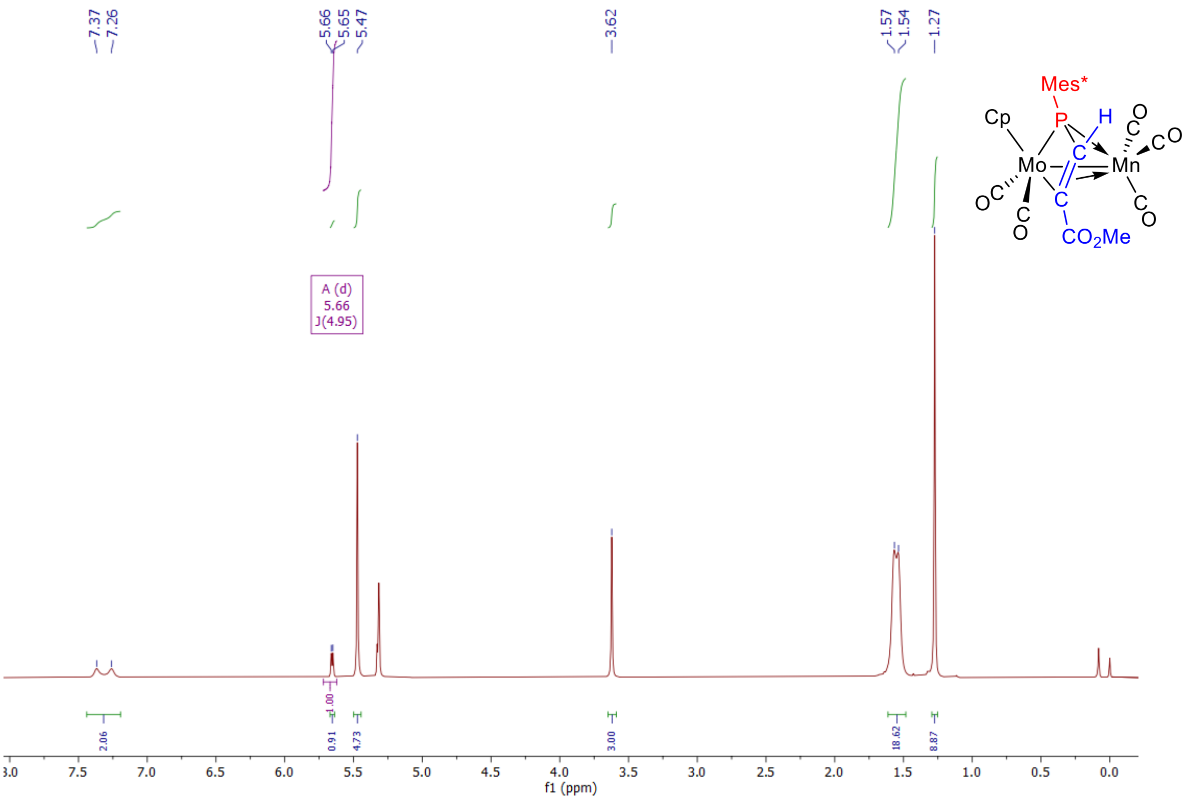

Figure S19. ^{1}H NMR spectrum of compound **3b.1** (CD_2Cl_2).

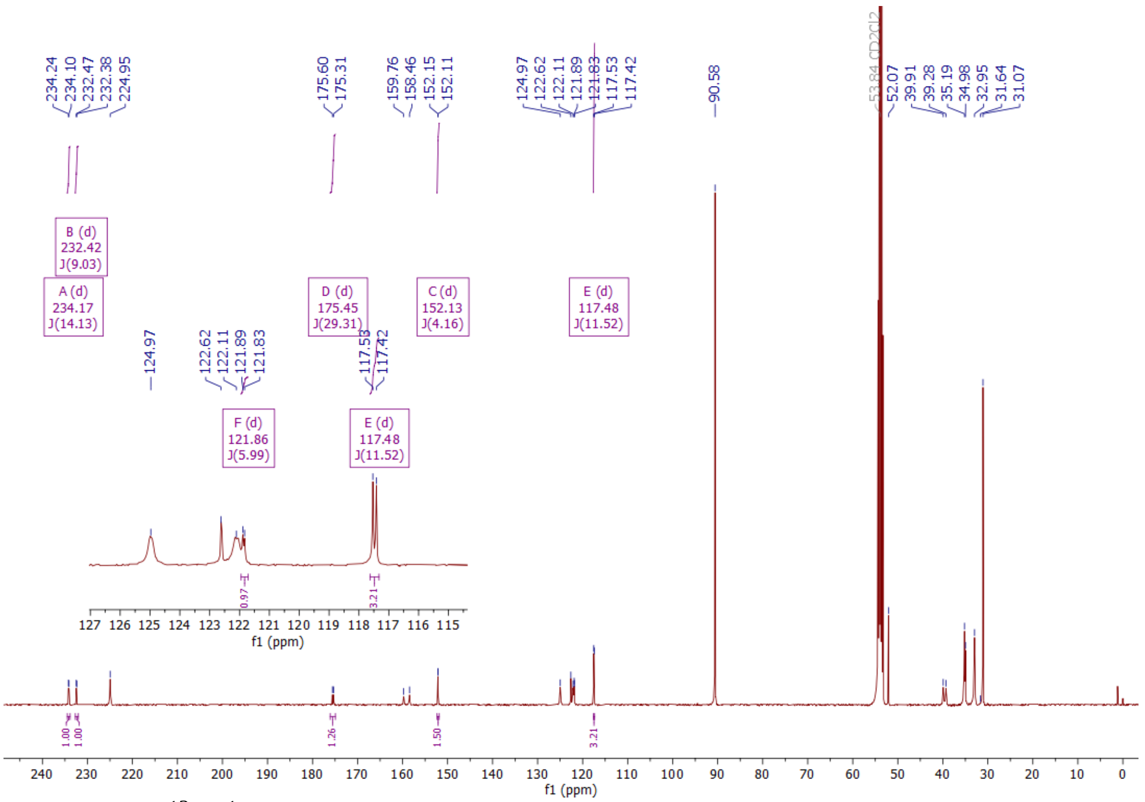

Figure S20. ^{13}C{^{1}H} NMR spectrum of compound **3b.1** (CD_2Cl_2).

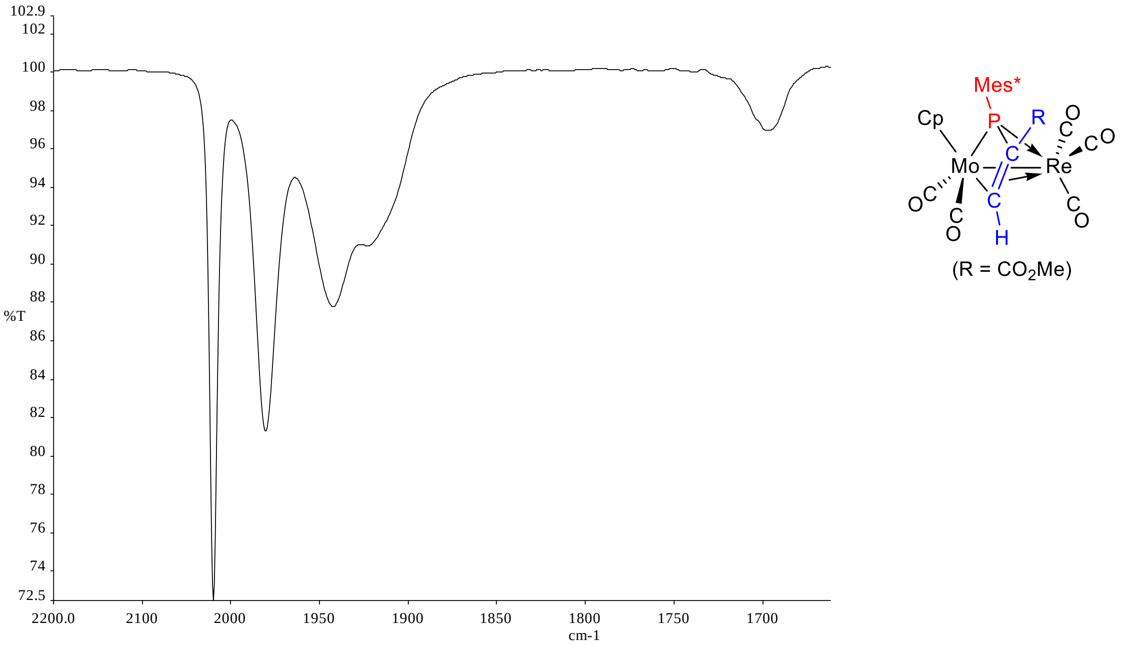

Figure S21. IR spectrum of compound **4b.1** in dichloromethane solution.

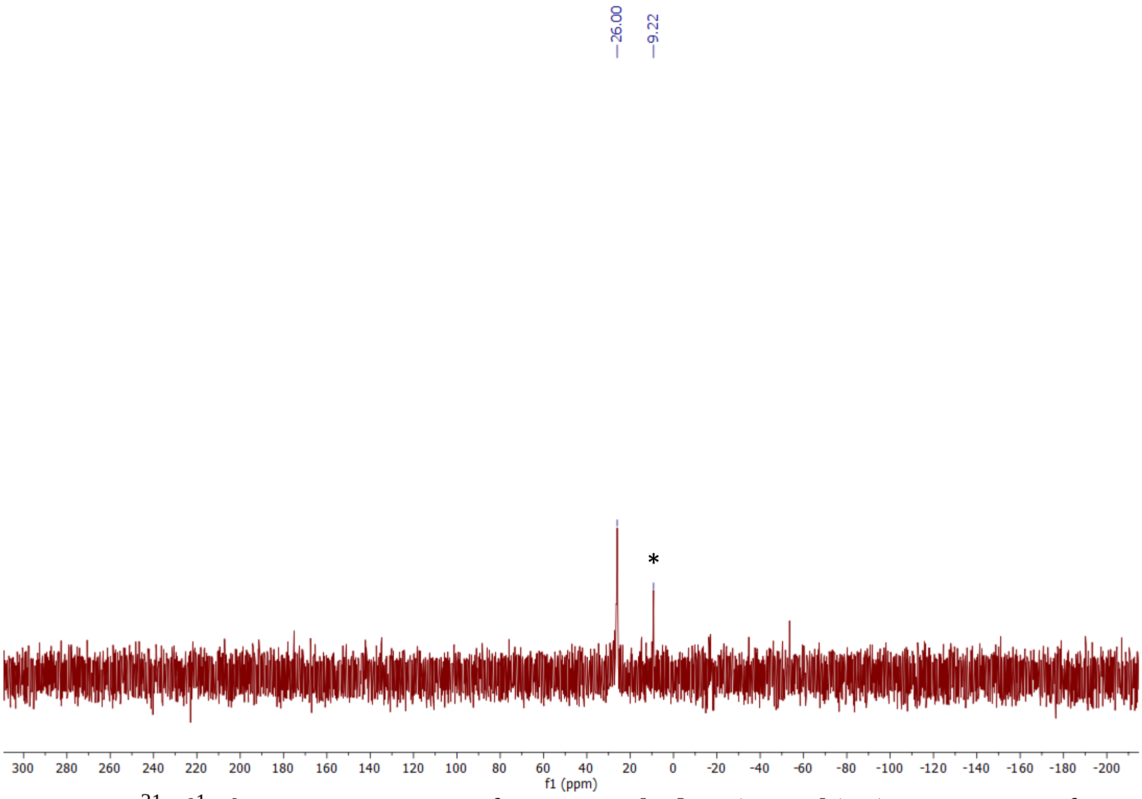

Figure S22. $^{31}P\{^{1}H\}$ NMR spectrum of compound **4b.1** (CD_2Cl_2). (***** resonance of isomer **3b.1**)

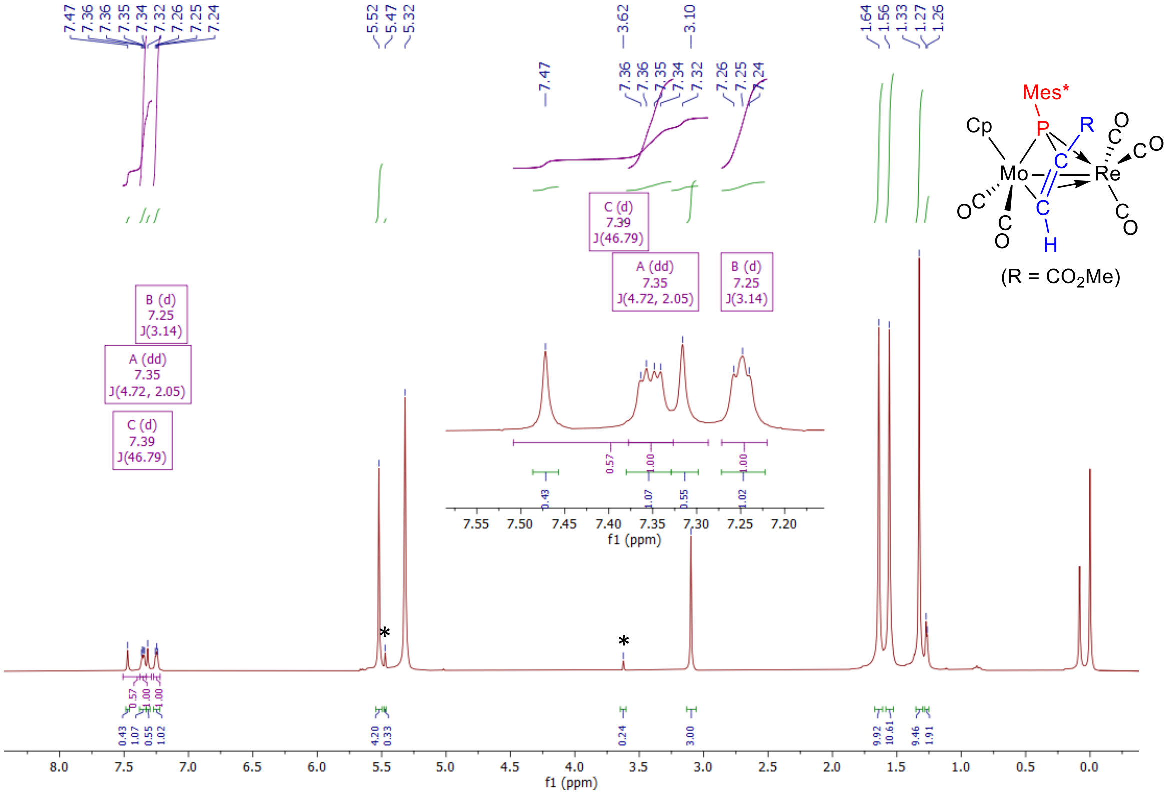

Figure S23. ^{1}H NMR spectrum of compound **4b.1** (CD_2Cl_2). (***** resonances of isomer **3b.1**)

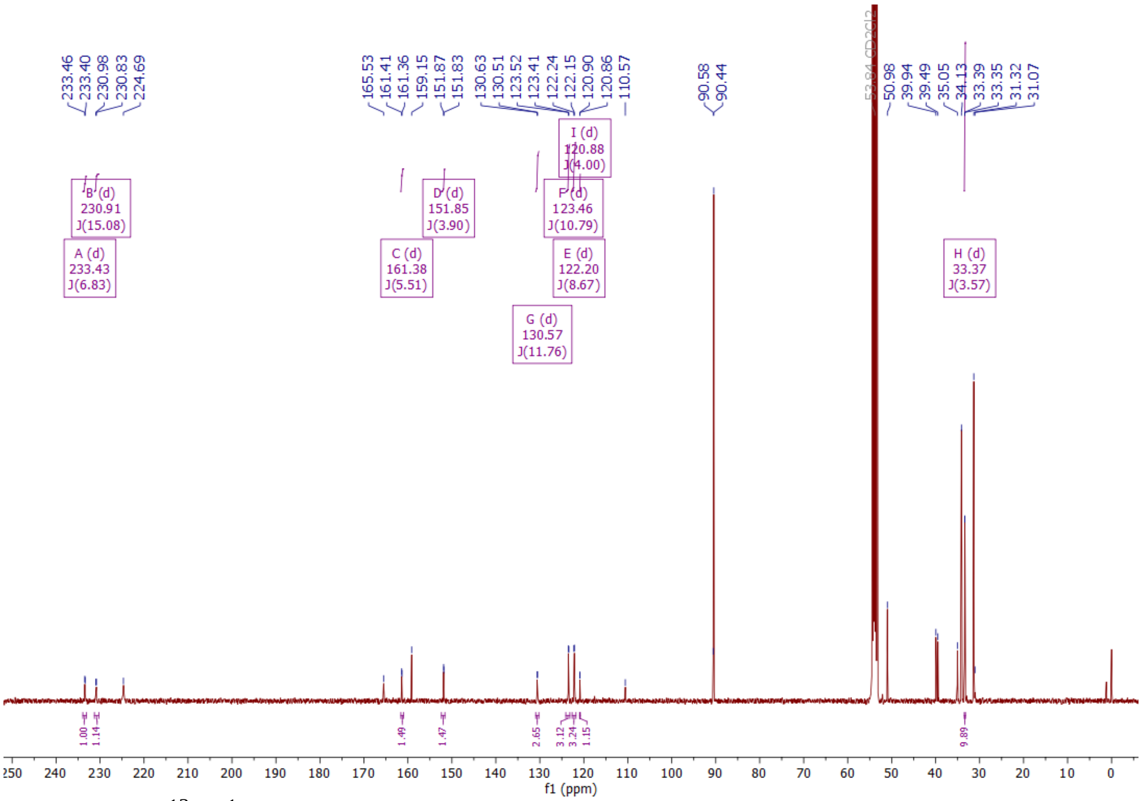

Figure S24. $^{13}C\{^{1}H\}$ NMR spectrum of compound **4b.1** (CD_2Cl_2).

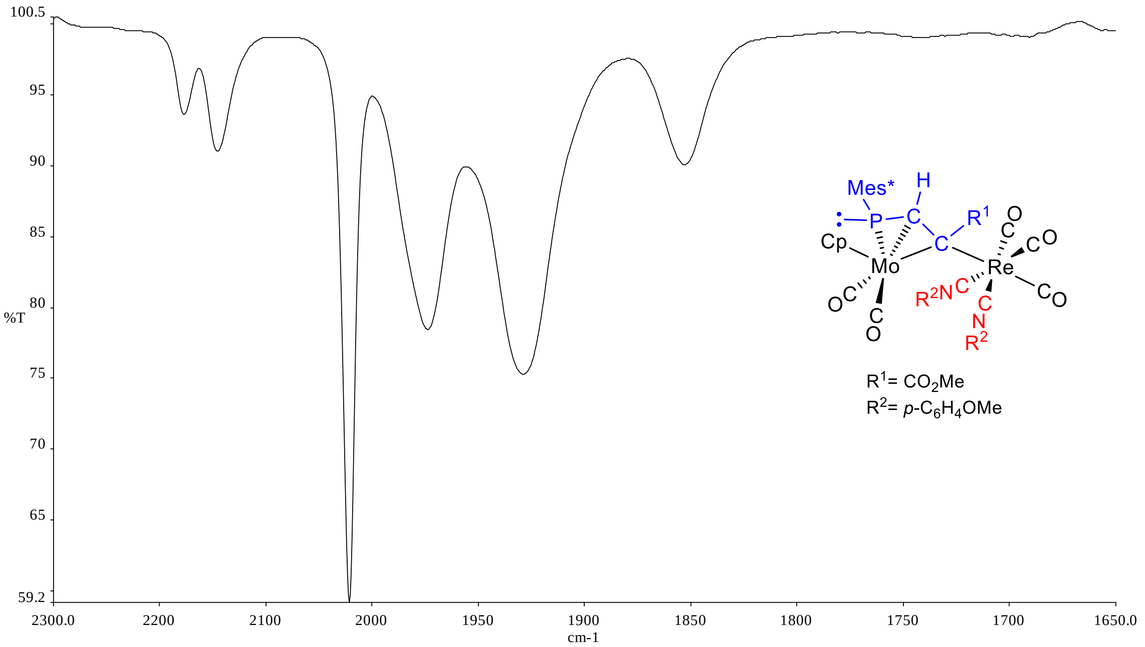

Figure S25. IR spectrum of compound **5** in dichloromethane solution.

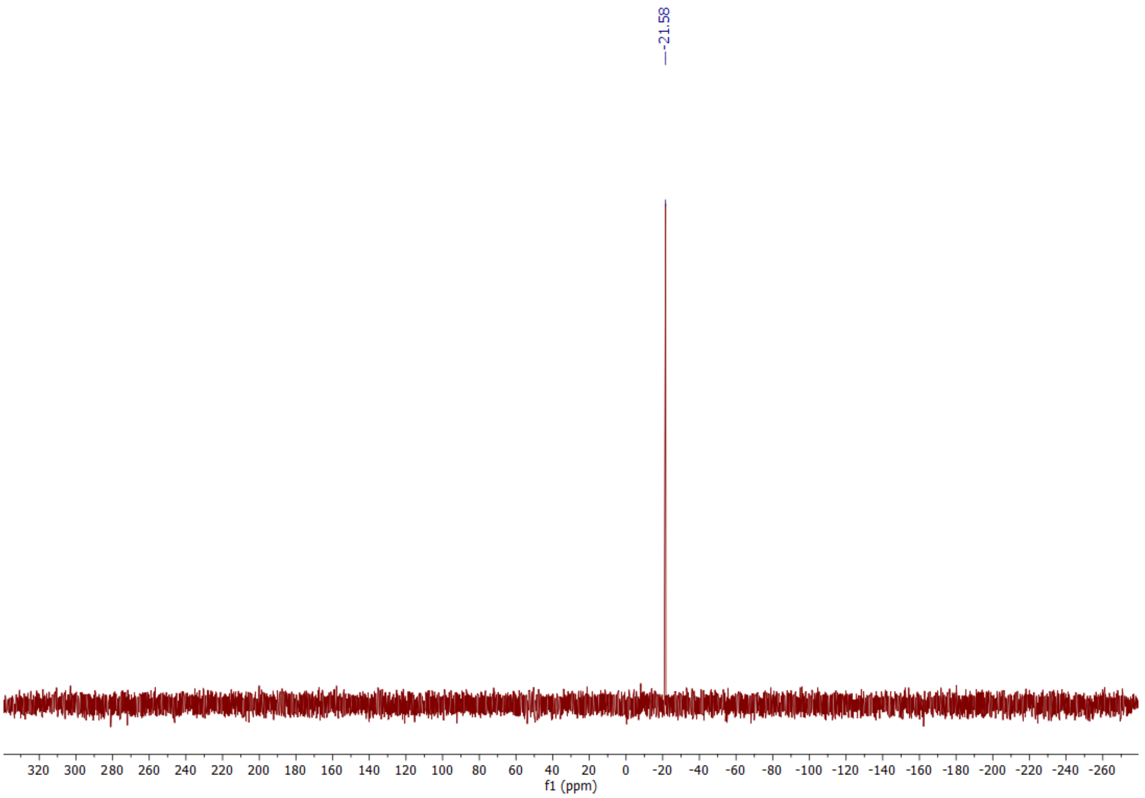

Figure S26. $^{31}P\{^{1}H\}$ NMR spectrum of compound **5** (CD_2Cl_2).

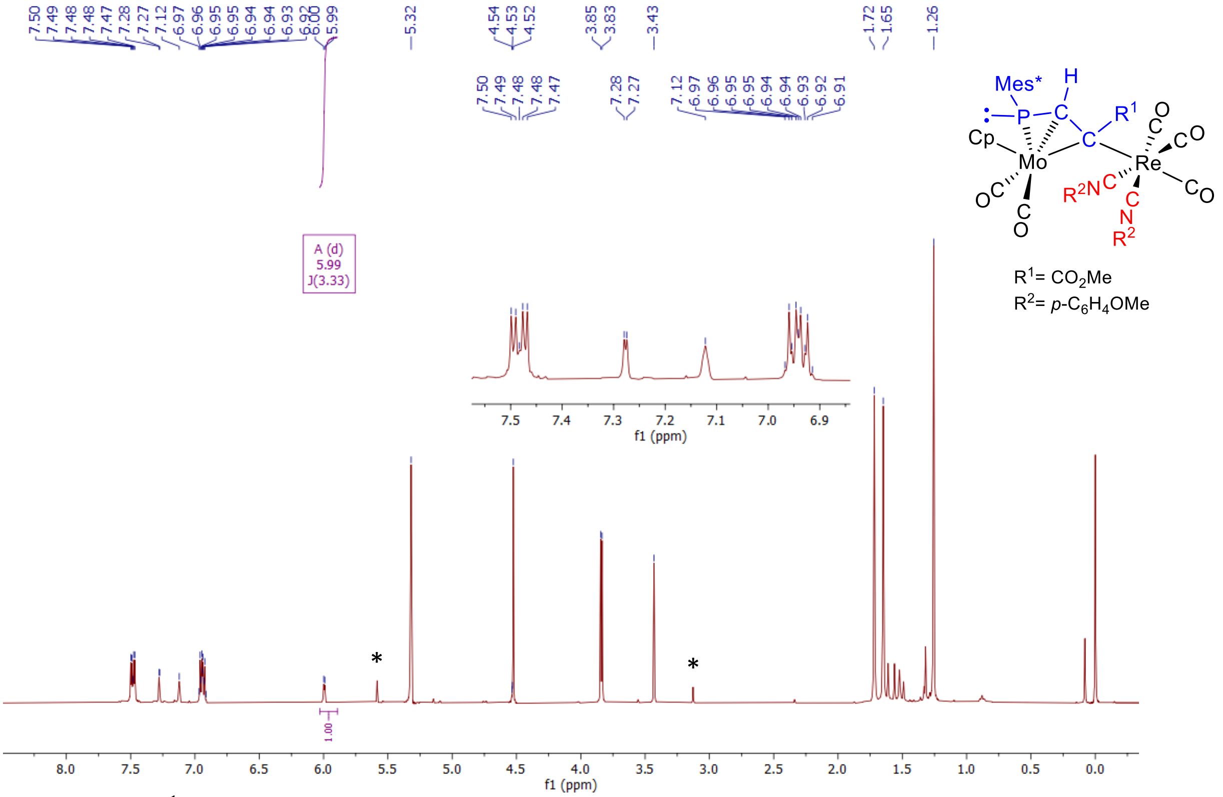

Figure S27. ^{1}H NMR spectrum of compound **5** (CD_2Cl_2). (***** resonances of compound **4a.1**)

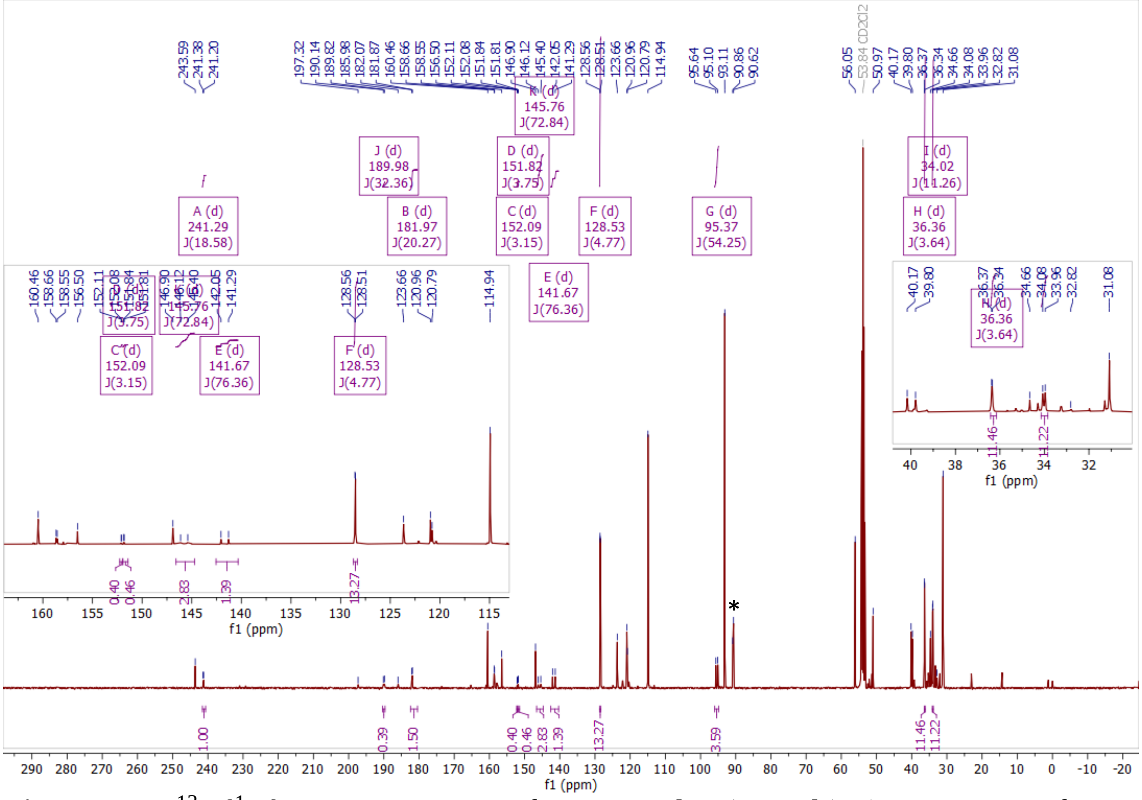

Figure S28. ^{13}C{^{1}H} NMR spectrum of compound **5** (CD_2Cl_2). (***** resonance of compound **4a.1**)

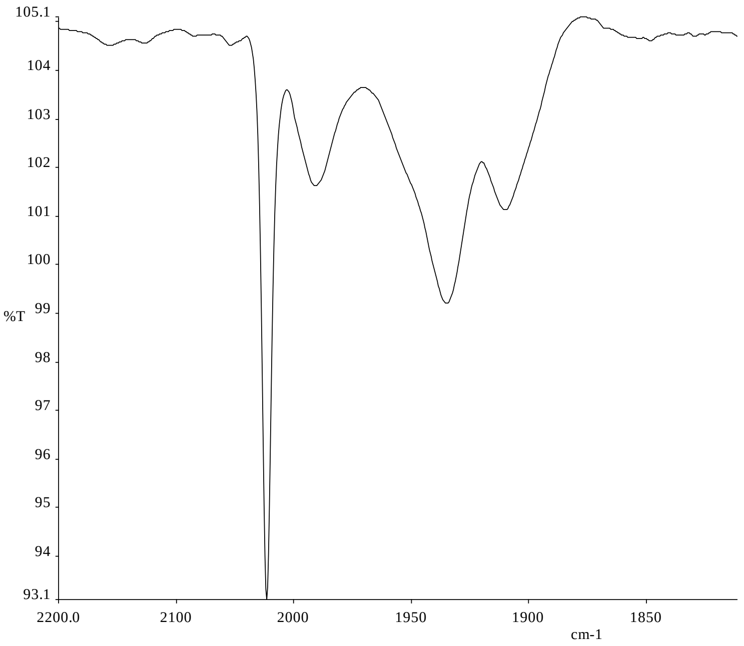

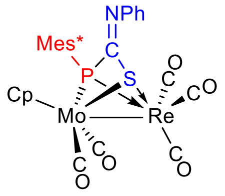

Figure S29. IR spectrum of compound **6** in dichloromethane solution.

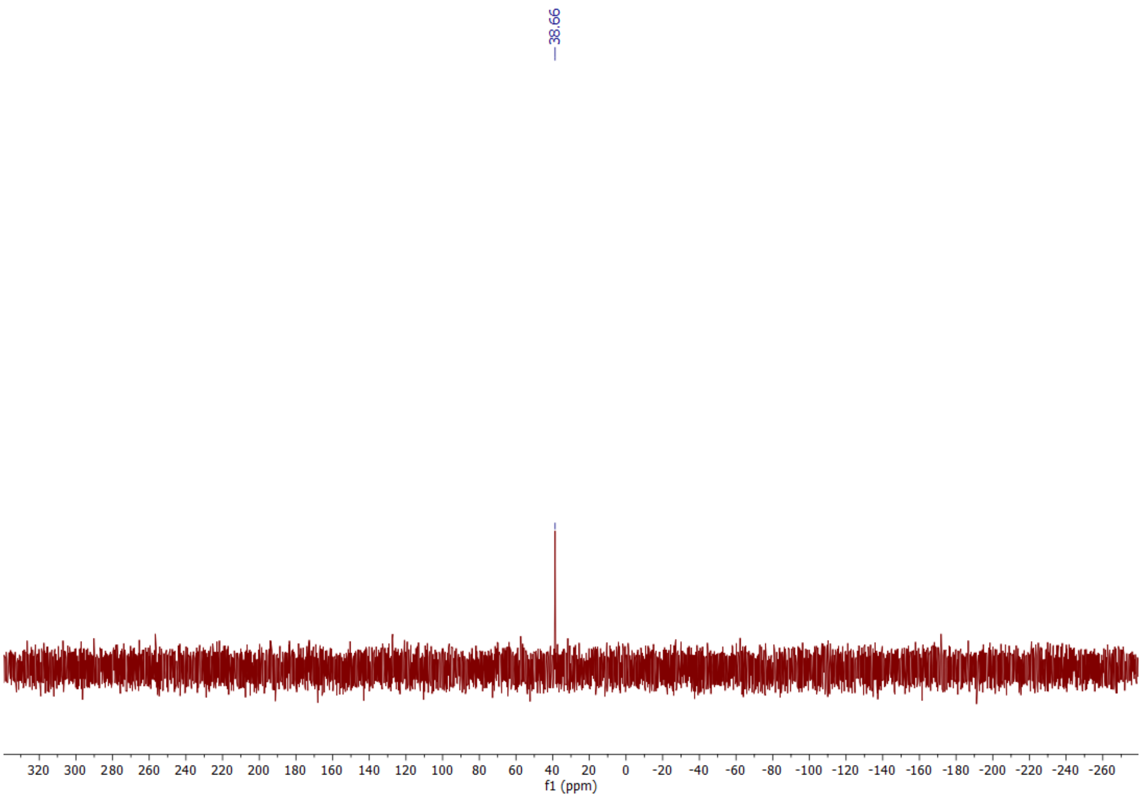

Figure S30. $^{31}P\{^{1}H\}$ NMR spectrum of compound **6** (CD_2Cl_2).

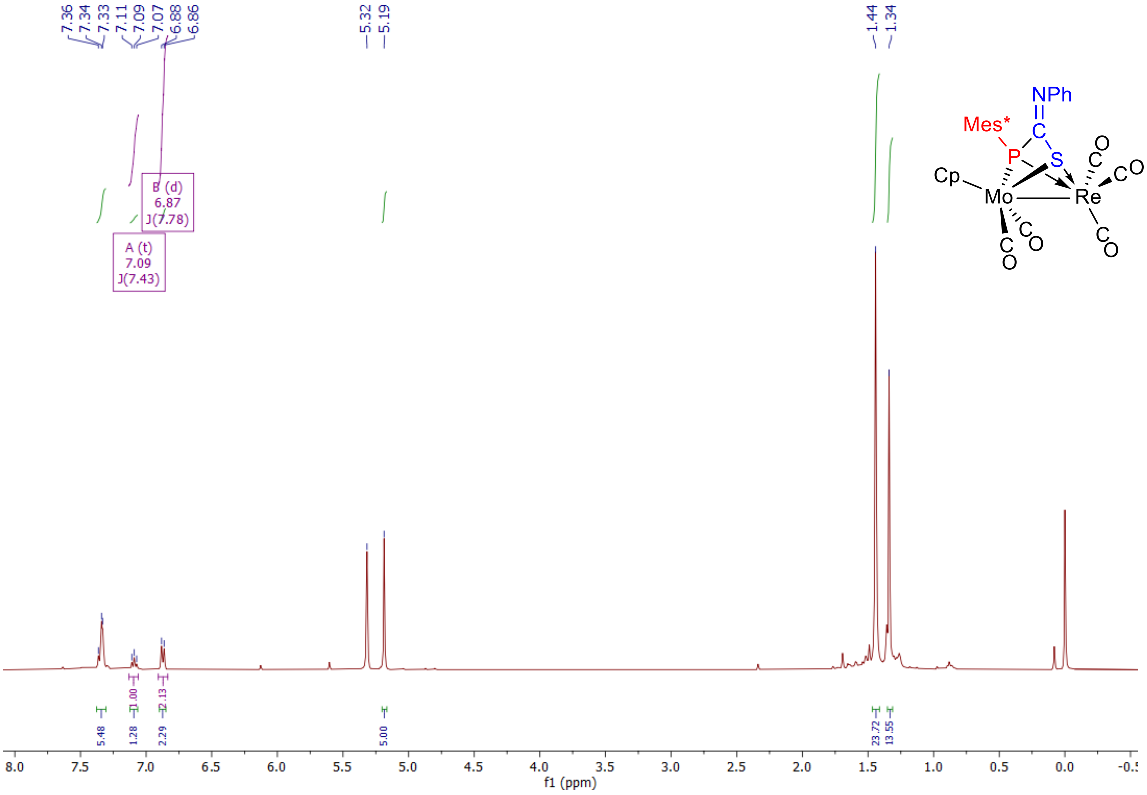

Figure S31. ^{1}H NMR spectrum of compound **6** (CD_2Cl_2).

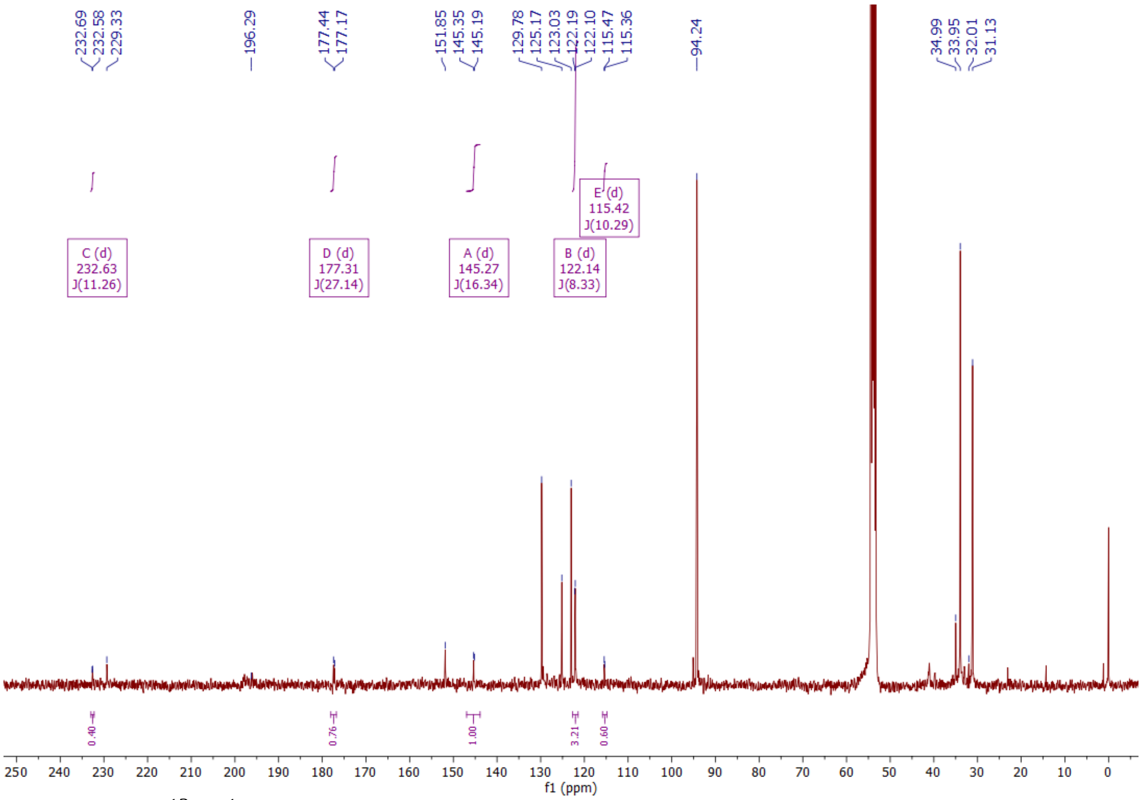

Figure S32. $^{13}C\{^{1}H\}$ NMR spectrum of compound **6** (CD_2Cl_2).

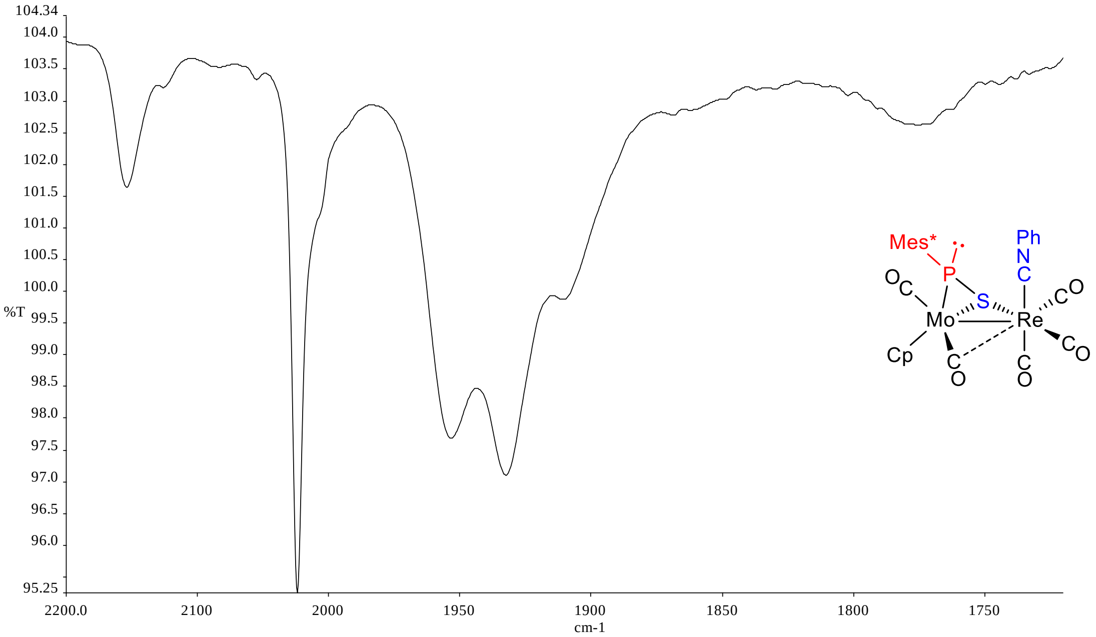

Figure S33. IR spectrum of compound **7** in dichloromethane solution.

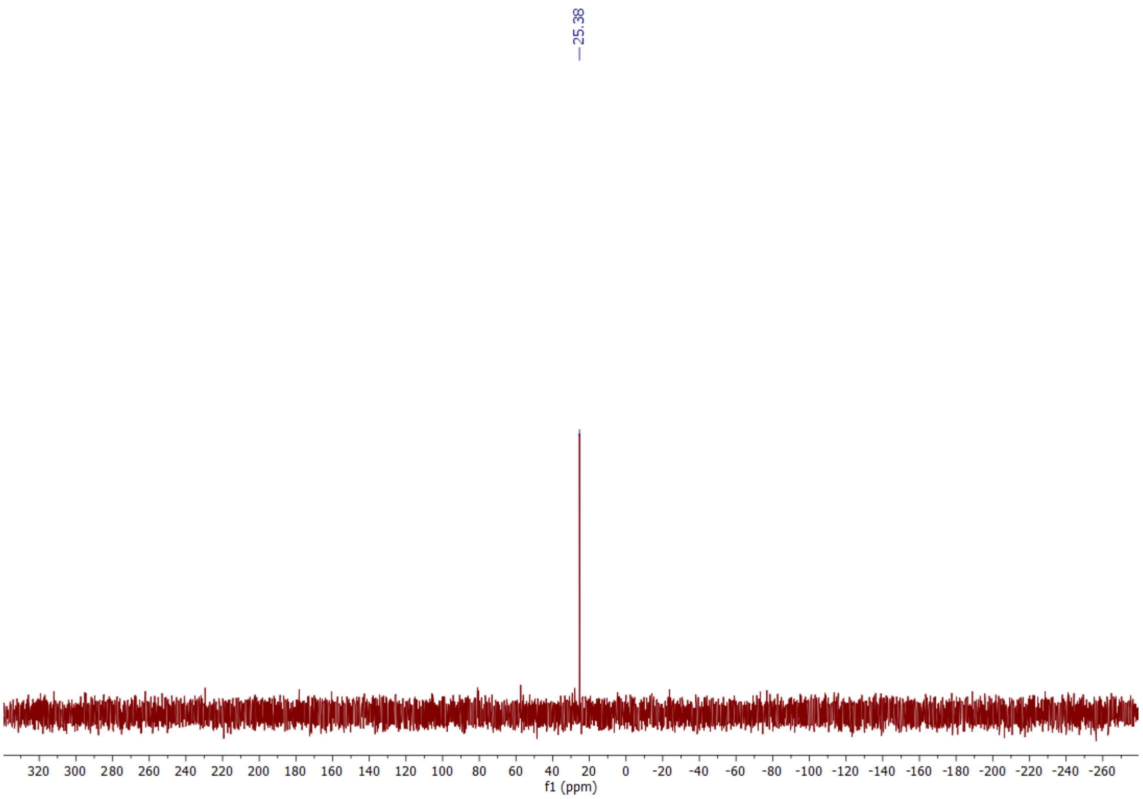

Figure S34. $^{31}P\{^{1}H\}$ NMR spectrum of compound **7** (CD_2Cl_2).

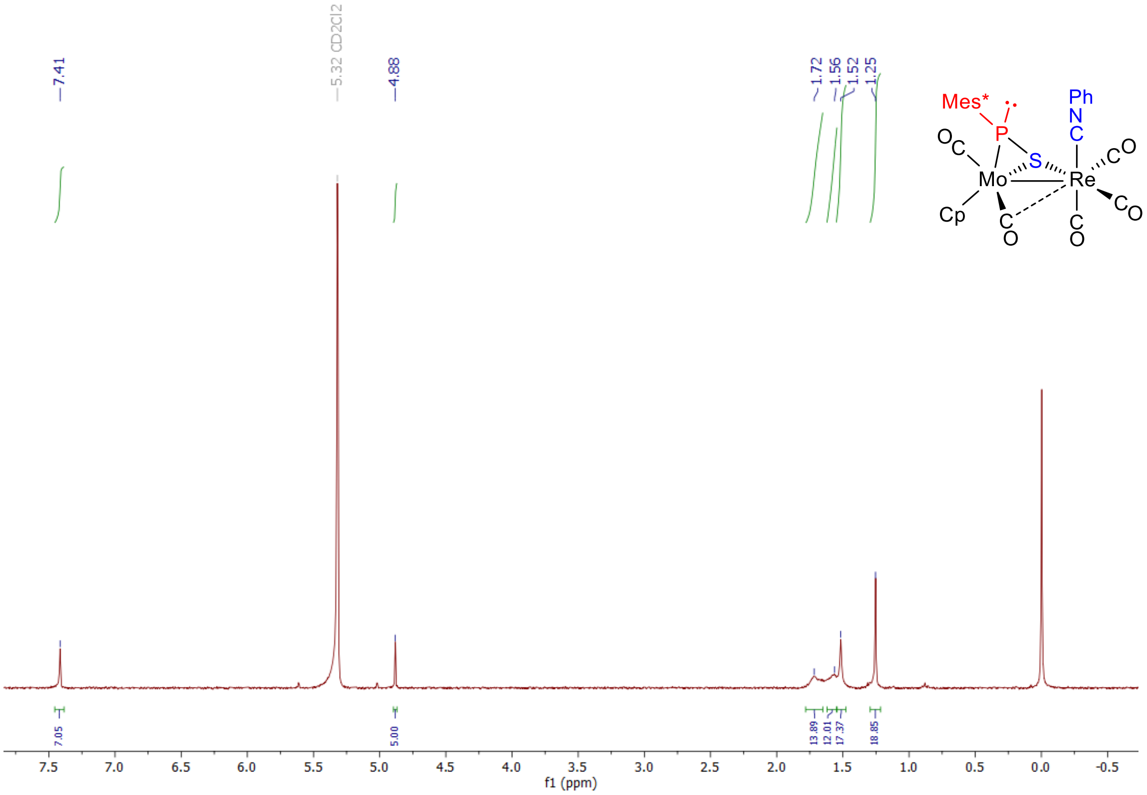

Figure S35. ^{1}H NMR spectrum of compound **7** (CD_2Cl_2).

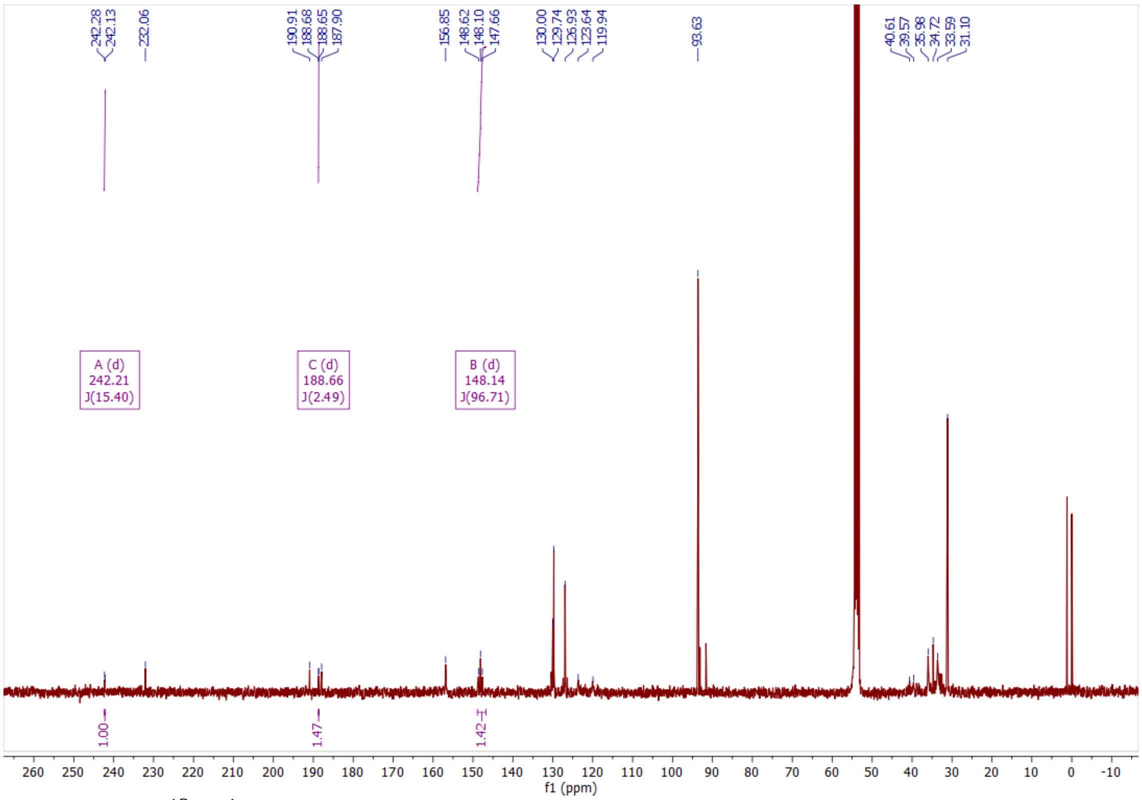

Figure S36. $^{13}C\{^{1}H\}$ NMR spectrum of compound **7** (CD_2Cl_2).

Figure S37. M06L-DFT optimized structures of compound **7** and related isomers and analogues, with H atoms omitted, with their Gibbs free energies at 295 K (in kJ/mol) indicated below, relative to the μ-κ^1_S:η^2-SPR isomer in each case.

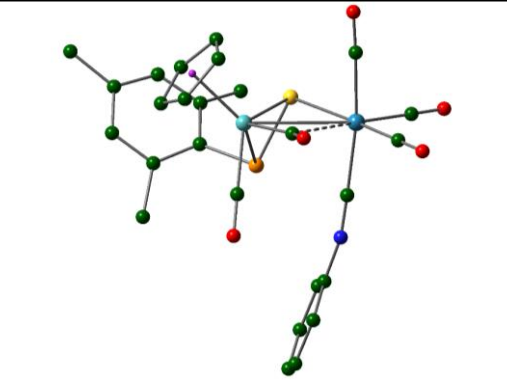	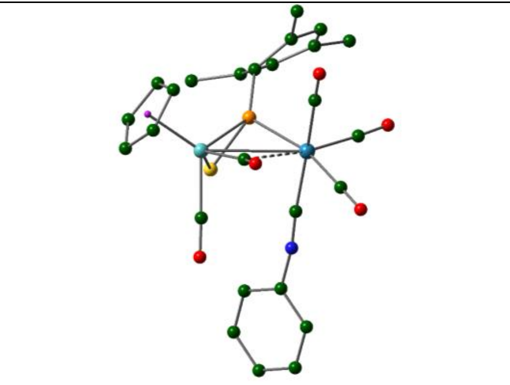
**7** (0)	***syn*-7P** (+77)
	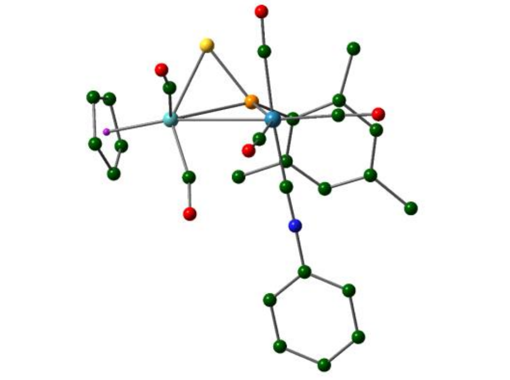
	***anti*-7P** (+61)
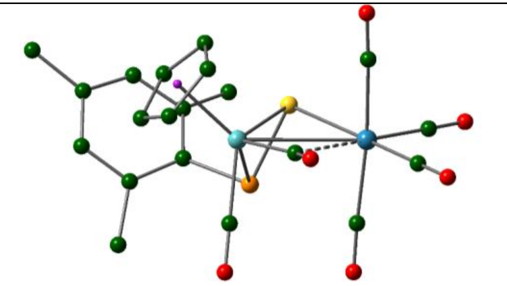	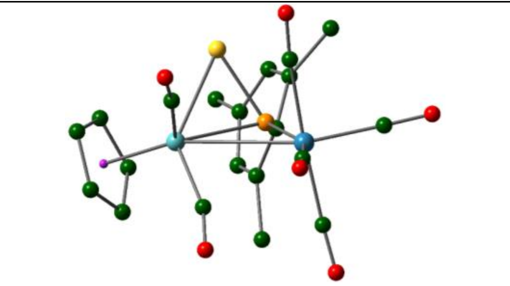
**8** (0)	**8-P** (+29)
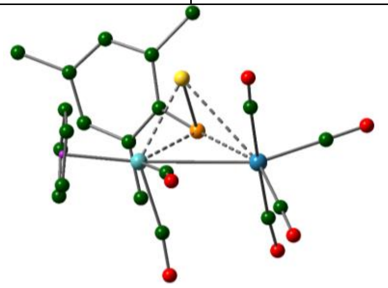	
**TS1** (+91)	

<table>
<tr><td>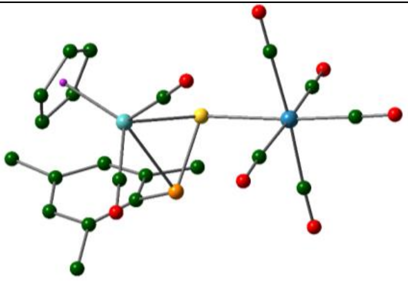</td><td>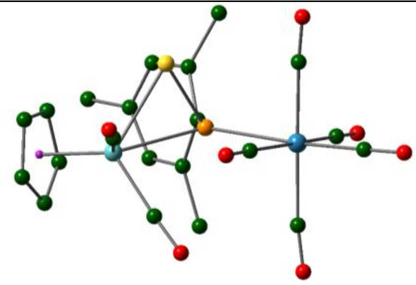</td></tr>
<tr><td>9 (0)</td><td>9-P (–6)</td></tr>
<tr><td colspan="2">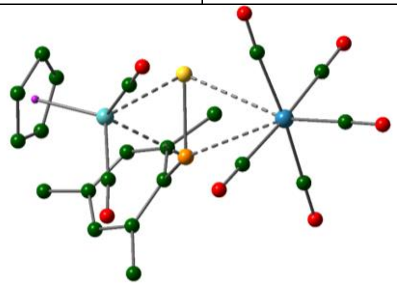</td></tr>
<tr><td colspan="2">TS2 (+55)</td></tr>
</table>
